# SARS-CoV-2 antigen rapid detection tests: test performance during the COVID-19 pandemic and the impact of COVID-19 vaccination

**DOI:** 10.1016/j.ebiom.2024.105394

**Published:** 2024-10-10

**Authors:** Isabell Wagenhäuser, Kerstin Knies, Tamara Pscheidl, Michael Eisenmann, Sven Flemming, Nils Petri, Miriam McDonogh, Agmal Scherzad, Daniel Zeller, Anja Gesierich, Anna Katharina Seitz, Regina Taurines, Ralf-Ingo Ernestus, Johannes Forster, Dirk Weismann, Benedikt Weißbrich, Johannes Liese, Christoph Härtel, Oliver Kurzai, Lars Dölken, Alexander Gabel, Manuel Krone

**Affiliations:** a Infection Control and Antimicrobial Stewardship Unit, University Hospital Würzburg, Josef-Schneider-Str. 2, 97080 Würzburg, Germany; b Department of Internal Medicine I, University Hospital Würzburg, Oberdürrbacher Str. 6, 97080 Würzburg, Germany; c Institute for Virology and Immunobiology, Julius-Maximilians-Universität Würzburg, Versbacher Str. 7, 97078 Würzburg, Germany; d Department of Anaesthesia and Critical Care, University Hospital Würzburg, Oberdürrbacher Str. 6, 97080 Würzburg, Germany; e Department of General, Visceral, Transplantation, Vascular, and Paediatric Surgery, University Hospital Würzburg, Oberdürrbacher Str. 6, 97080 Würzburg, Germany; f Department of Orthopaedic Trauma, Hand, Plastic, and Reconstructive Surgery, University Hospital Würzburg, Oberdürrbacher Str. 6, 97080 Würzburg, Germany; g Department of Otorhinolaryngology, Plastic, Aesthetic, and Reconstructive Head and Neck Surgery, University Hospital Würzburg, Josef-Schneider-Str. 11, 97080 Würzburg, Germany; h Department of Neurology, University Hospital Würzburg, Josef-Schneider-Str. 11, 97080 Würzburg, Germany; i Department of Dermatology, Venerology, and Allergology, University Hospital Würzburg, Josef-Schneider-Str. 2, 97080 Würzburg, Germany; j Department of Urology, University Hospital Würzburg, Oberdürrbacher Str. 6, 97080 Würzburg, Germany; k Department of Child and Adolescent Psychiatry, Psychosomatics, and Psychotherapy, University Hospital Würzburg, Margarete-Höppel-Platz 1, 97080 Würzburg, Germany; l Department of Neurosurgery, University Hospital Würzburg, Josef-Schneider-Str. 11, 97080 Würzburg, Germany; m Institute for Hygiene and Microbiology, Julius-Maximilians-Universität Würzburg, Josef-Schneider-Str. 2, 97080 Würzburg, Germany; n Department of Paediatrics, University Hospital Würzburg, Josef-Schneider-Str. 2, 97080 Würzburg, Germany; o Leibniz Institute for Natural Product Research and Infection Biology – Hans-Knoell-Institute, Beutenbergstraße 13, 07745 Jena, Germany; p Helmholtz Institute for RNA-based Infection Research (HIRI), Helmholtz Centre for Infection Research (HZI), Josef-Schneider-Str. 2, 97080 Würzburg, Germany

**Keywords:** SARS-CoV-2 rapid antigen detection test, Test performance, COVID-19 vaccination, Virus variants of concern, COVID-19 symptoms, Viral load

## Abstract

**Background:**

SARS-CoV-2 antigen rapid detection tests (RDTs) emerged as point-of-care diagnostics alongside reverse transcription polymerase chain reaction (RT-qPCR) as reference.

**Methods:**

In a prospective performance assessment from 12 November 2020 to 30 June 2023 at a single centre tertiary care hospital, the sensitivity and specificity (primary endpoints) of RDTs from three manufacturers (NADAL®, Panbio™, MEDsan®) were compared to RT-qPCR as reference standard among patients, accompanying persons and staff aged ≥ six month in large-scale, clinical screening use. Regression models were used to assess influencing factors on RDT performance (secondary endpoints).

**Findings:**

Among 78,798 paired RDT/RT-qPCR results analysed, overall RDT sensitivity was 34.5% (695/2016; 95% CI 32.4–36.6%), specificity 99.6% (76,503/76,782; 95% CI 99.6–99.7%). Over the pandemic course, sensitivity decreased in line with a lower rate of individuals showing typical COVID-19 symptoms. The lasso regression model showed that a higher viral load and typical COVID-19 symptoms were directly significantly correlated with the likelihood of a positive RDT result in SARS-CoV-2 infection, whereas age, sex, vaccination status, and the Omicron VOC were not.

**Interpretation:**

The decline in RDT sensitivity throughout the pandemic can primarily be attributed to the reduced prevalence of symptomatic infections among vaccinated individuals and individuals infected with Omicron VOC. RDTs remain valuable for detecting SARS-CoV-2 in symptomatic individuals and offer potential for detecting other respiratory pathogens in the post-pandemic era, underscoring their importance in infection control efforts.

**Funding:**

10.13039/501100002347German Federal Ministry of Education and Research (BMBF), Free State of Bavaria, Bavarian State Ministry of Health and Care.

**Research in context**

**Evidence before this study**

We searched PubMed® using the following search terms: ((“COVID-19”) OR (“COVID”) OR (“SARS-CoV-2”) OR (“coronavirus”)) AND ((“antigen detection”) OR (“rapid antigen test”) OR (“Point-of-care test”)) AND ((“COVID-19 vaccination”) OR (“VOC”) OR (“VOI”) OR (“variant”) OR (“virus variant of concern”) OR (“viral load”) OR (“symptoms”) OR (“test performance”)) published between 1 January 2020 and 25 December 2023.

To date, a large body of evidence has demonstrated in detail that RDT sensitivity and specificity can be far below the manufacturer's specifications and do not correspond to the gold standard of RT-qPCR although most of the evidence to date does not cover the entire COVID-19 pandemic, analyses only small, selective test collectives or does not evaluate RDTs in screening use. The following correlations have already been proven in the evidence to date as decisive factors influencing test performance: the presence of typical COVID-19 symptoms and high viral load correlate positively with high RDT sensitivity. Evidence to date is heterogeneous that the Omicron VOC might reduce the RDT performance. Data from an interim analysis of the study presented suggests that any deterioration in RDT performance is not attributable to VOC itself but rather to the change in symptomatology mediated by the VOC throughout the course of the COVID-19 pandemic. The role of COVID-19 vaccination was not incorporated.

Further, regarding the potential influence of COVID-19 vaccination on RDT performance only very few studies have so far considered the aspect of COVID-19 vaccination. One study discussed the hypothesis that the observed decrease in RDT sensitivity in clinical use, despite higher viral loads, is attributable to increased immunity among the study population due to COVID-19 vaccinations and previous SARS-CoV-2 infections. In contrast, two other studies that consider the potential influence of COVID-19 vaccination status on the large-scale clinical RDT test performance factor do not observe any impact of vaccination status on RDT performance. However, all three only cover the pandemic period up to early 2022.

**Added value of this study**

The study investigates RDT performance compared to RT-qPCR with standardised viral load in a large clinical cohort encompassing the entire COVID-19 pandemic, considering the possible influencing factors of RDT performance, foremost among them being SARS-CoV-2 VOC and COVID-19 vaccination status. It could be comprehensively demonstrated based on a lasso regression analysis that only two factors, viral load, and COVID-19 symptom status, directly significantly influence RDT performance while vaccinations influence RDT significantly negatively by reducing the frequency of typical symptomatic infections.

**Implications of all the available evidence**

The present study offers a comprehensive analysis of how RDTs have performed throughout the COVID-19 pandemic, elucidating factors influencing their efficacy. COVID-19 vaccination and Omicron VOC indirectly affect RDT performance negatively mediated by a reduced frequency of typical symptomatic infections. RDTs prove reliable in detecting SARS-CoV-2 when typical respiratory symptoms are present independently of vaccination status and VOC, though RT-qPCR remains the gold standard. Moreover, their versatility extends beyond SARS-CoV-2, as evidenced by their adaptability for other pathogens like Influenza and RSV.

## Introduction

During the COVID-19 pandemic, timely, rapid, and reliable diagnosis of SARS-CoV-2 were a cornerstone of efforts to reduce the virus' spread.^1^ The acute phase of the pandemic has now passed, and COVID-19 is in transition as a seasonal pathogen of acute respiratory diseases.^2^

As a well-established, very precise method, reverse transcription polymerase chain reaction (RT-qPCR) has been the gold standard for diagnostics since the beginning of the pandemic. For a more rapid, cost-effective and point-of-care diagnostics, SARS-CoV-2 antigen rapid detection tests (RDT) were made available as lateral flow immunoassays just a few months after the beginning of the pandemic without infrastructural requirements and rapid results, but performance limitations compared to RT-qPCR.^3^, ^4^, ^5^, ^6^, ^7^, ^8^, ^9^, ^10^

However, since 2020, many circumstances in the test environment have changed which requires the re-evaluation of RDT performance under this current conditions. With the course of the pandemic, the infestation of society and the availability of COVID-19 vaccines, there is now a basic immunised test collective.^11^ As the various SARS-CoV-2 virus variants of concern (VOC) progressed, the initial wild-type SARS-CoV-2 was chronologically displaced first by the Alpha and Delta VOC and ultimately by the Omicron VOC with its various sublines. This paved the way from pandemic to endemic with lower morbidity and a population that was immunised in parallel by previous infections and the available vaccinations.^12^ The current evidence that can assess those influencing factors as a whole in a large cohort in clinical application, however, either does not consider all possible factors, is not peer-reviewed, or only covers the COVID-19 pandemic until early 2022.^7^, ^8^, ^9^^,^^13^, ^14^, ^15^

The study analyses the large-scale RDT test performance and its influencing factors in clinical screening use, especially the role of COVID-19 vaccination and SARS-CoV-2 VOC, in the longitudinal course of the COVID-19 pandemic until its endemic transition in 2023.

## Methods

### Study design

This study is a prospective performance assessment conducted at a single tertiary care hospital from November 12, 2020, to June 30, 2023. The study aimed to evaluate the diagnostic accuracy of SARS-CoV-2 RDTs from three manufacturers (NADAL®, Panbio™, MEDsan®) in comparison to the RT-qPCR reference standard.^16^, ^17^, ^18^

The primary endpoints were the sensitivity and specificity of the RDTs. Sensitivity was defined as the proportion of true positive RDT results among all RT-qPCR-confirmed positive cases, while specificity was defined as the proportion of true negative RDT results among all RT-qPCR-confirmed negative cases.

Secondary endpoints included factors influencing RDT performance, assessed using regression models. These factors comprised viral load, COVID-19 symptoms, age, sex, vaccination status, and VOC. These factors were selected because their influence on test performance is discussed in the literature, and they can potentially impact the testing strategy.^3^^,^^4^^,^^9^^,^^10^^,^^13^

The study population included patients, accompanying persons, and staff aged six months and older, who were part of large-scale clinical screening efforts within a German 1438-bed tertiary care hospital.

The detailed strategy for implementing RDT throughout the clinic and the RDT deployment strategy adapted to the various phases of the pandemic are described in the Supplementary Methods.

### Data collection

The following inclusion criteria were defined for considering a paired RDT/RT-qPCR result for the analysis:

- documented RDT with parallel RT-qPCR
- valid test result of the RDT (presence of a control line, no interference lines)
- age ≥ six month

The age limit of ≥ six months was implemented given that the European Medicines Agency (EMA) allowed COVID-19 vaccination only for individuals aged ≥ six months.^19^, ^20^, ^21^, ^22^

Documented RDTs were excluded from data analysis in the following situations:

- multiple testing (more than one RDT per day and person): only the first chronologically performed RDT per day and person were considered. Patients meeting the inclusion criteria on multiple days during the study period underwent testing and inclusion once per visit.
- recent SARS-CoV-2 infections and subsequent deisolation were excluded from the analysis due to the potential persistence of RT-qPCR positivity unrelated to the risk of viral transmission.^23^

**Fig. 1:**
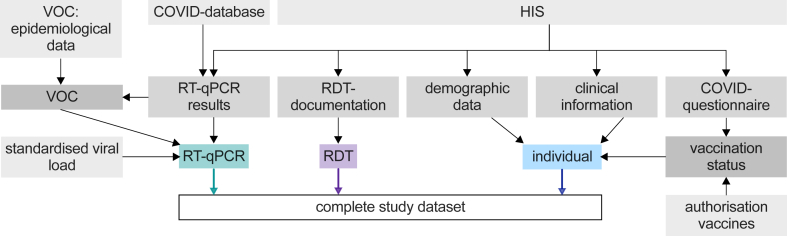
Schematic overview of data acquisition. HIS: hospital information system. RDT: Antigen rapid detection test. RT-qPCR: Quantitative reverse transcription-polymerase chain reaction. VOC: SARS-CoV-2 virus variant of concern.

For RDT and RT-qPCR testing the swabs were taken as paired, consecutively collected oropharyngeal samples by trained healthcare workers. All the manufacturer's instructions were conscientiously followed with the single deviation that the Panbio™ RDT was performed with oropharyngeal instead of recommended nasopharyngeal swabs.^17^ The overall dataset was merged from the following sources (Fig. 1):

- hospital information system (HIS; SAP ERP 6.0 (SAP, Walldorf, Germany)): RDT documentation, RT-qPCR results, demographic data, clinical information, information on COVID-19 vaccination
- hospital's COVID-database with a systematic overview about all positive SARS-CoV-2 detections at the hospital
- epidemiological data on VOC prevalence in Germany^12^
- standardised viral load calculation of the RT-qPCR positive samples
- EMA COVID-19 vaccination authorisation data^19^, ^20^, ^21^, ^22^

According to Bavarian State law (Art. 27 BayKrG, Bavarian Hospital Act), no explicit informed consent was necessary as the anonymized data used for this study.

Subjects were classified based COVID-19 case definition provided by the ECDC^24^ into the following cohorts:

- typical COVID-19 symptoms: individuals suffering fever, dry cough, shortness of breath, new anosmia, or ageusia
- atypical COVID-19 symptoms potentially be linked to COVID-19: individuals with a decline in general condition, falls, diarrhoea, or seizures
- asymptomatic individuals

The vaccination status of the patients at the time of each RDT was determined by evaluating the admission questionnaire incorporating the official EMA approval data of COVID-19 vaccines (Supplementary Methods).^19^, ^20^, ^21^, ^22^

### Antigen rapid detection tests (RDT)

To maintain an uninterrupted logistical provision, three specific RDT were chosen from a pool of 23 products identified by the German Federal Institute for Drugs and Medical Devices in October 2020.^5^^,^^25^ All the RDTs used are listed on the EU Common List of COVID-19 antigen tests by the European commission (directorate-general for health and food safety).^26^

(I) NADAL® COVID-19 Ag Test (Nal von Minden GmbH, Regensburg, Germany)^16^
(II) Panbio™ COVID-19 Ag Rapid Test (Abbott Laboratories, Abbott Park IL, USA)^17^
(III) MEDsan® SARS-Cov-2 Antigen Rapid Test (MEDsan GmbH, Hamburg, Germany)^18^

All three RDTs used for the study are designed as lateral flow immunoassays with the SARS-CoV-2 nucleoprotein antigen as the target structure, according to manufacturer information. NADAL® and MEDsan® RDTs are approved for use with oropharyngeal swabs.^16^^,^^18^ The Panbio™ RDT is approved for nasopharyngeal swabs, but in this study, it was also used with oropharyngeal swabs in comparison to RT-qPCR.^17^

The distribution of RDTs to the individual hospital's departments was random, depending on availability, independent of the current RDT deployment concept. All RDTs performed as part of the study were carried out directly point-of-care, decentralised immediately after the swab, following manufacturer instructions by trained medical staff, and results were documented. Since RT-qPCR diagnostics were only available after the RDT processing time due to logistics and RT-qPCR processing time, the interpretation of the RDT was always done without knowledge of the RT-qPCR result.

### RT-qPCR and viral load determination

RT-qPCR diagnostics were processed in the hospitals’ virological diagnostic laboratories utilising several RT-qPCR methods adhering to the guidelines provided by the respective manufacturers. To prioritise RDT-positive samples for the fastest possible confirmation by RT-qPCR, the RDT results were made available to the virus diagnostics staff. The used RT-qPCR analytical instruments are described in the Supplementary Methods.

Viral loads were computed from C_t_-values employing the previously described formula with reference standards, as follows:^5^

$$ViralLoad\;\left(Sample\right)=ViralLoad\;\left(S_{1}\right)\;\times \;{\left(\sqrt[{C_{t}\left(S_{1}\right)-C_{t}\left(S_{2}\right)}]{\frac{\;ViralLoad\left(S_{2}\right)}{\;ViralLoad\left(S_{1}\right)}}\right)}^{\left(C_{t}\left(S_{1}\right)-C_{t}\left(Sample\right)\right)}$$

For instances involving multiple targets with distinct C_t_-values on an RT-qPCR system (cobas®, NeuMoDx™, Xpert® Xpress SARS-CoV-2/Flu/RSV), the viral load was determined by computing the geometric mean of the estimates derived from the two individual genes (Supplementary Figure S2).

### SARS-CoV-2 virus variant of concern

Between 3 February 2021 and 19 January 2022, for allocation of VOC of all RT-qPCR-positive samples with sufficient viral load a PCR with spike protein variant-specific differentiation was performed. Outside of the phase of molecular VOC determination, variant assignment was done epidemiologically wherever possible. The precise procedure of molecular and epidemiological VOC assignment is described in the Supplementary Methods and Supplementary Table S1.^11^^,^^12^

### Ethical approval

The Ethics committee of the University of Würzburg considered the study protocol and waived the need to formally apply for ethical clearance due to the study design (File Nr 20231219 02).

### Statistics

The data in the overall RDT dataset were recorded using Excel 2019 (Microsoft, Redmond WA, USA). The hospital's COVID-19 database is based on an Access 2019 (Microsoft, Redmond WA, USA) platform. Statistical analyses were conducted using GraphPad Prism 10.2.1 (GraphPad Software, San Diego CA, USA), and R (Version 4.1.3).

Confidence intervals were calculated using the Wilson-Brown method (RDT performance).^27^

Pairwise comparisons were performed to analyse differences in sensitivity by manufacturers, VOC, vaccination status, and symptoms using the Fisher–Exact test and differences in viral load by VOC, vaccination status, and symptoms using the Mann-Whitney-U test.

A logistic lasso regression was performed to identify factors associated with the RDT result confirming a SARS-CoV-2 infection as dependent variable. The regression model included the independent variables age, sex, manufacturer, viral load, typical COVID-19 symptoms, COVID-19 vaccination, and infection by the Omicron VOC. Using a tenfold cross-validation procedure, the model parameters of the Lasso regression model were estimated (Supplementary Figure S3). To assess a potential influence multiple inclusions of individuals.^28^ This analysis was repeated only including the first RDT/RT-qPCR pair of each individual (Supplementary Statistics). In order to avoid misinterpretation of the associated factors from a single model,^29^ we investigated separate logistic regression models for each individual factor, such as viral load, COVID-19 symptoms, COVID-19 vaccination, and infection by the Omicron VOC, as well as for combination of these factors. This approach allowed us to differentiate between the total effects of individual factors and total effects within combinations of factors.

An additional logistic lasso regression analysis was employed to investigate the influence of the following independent factors on the dependent variable typical COVID-19 symptoms: age, sex, VOC, and COVID-19 vaccination.

For both regression analyses, only those RDT/RT-qPCR results with available vaccination status and assigned VOC were considered. To correct against multiple testing, the resulting p-values were adjusted using the Benjamini-Yekutieli procedure.^30^

Adjusted p-values <0.05 were considered statistically significant.

### Role of the funding source

This study was initiated by the researchers themselves. The funding institutions had no influence on the study design, data collection, analysis and interpretation, or the writing of the manuscript. All authors had unrestricted access to all data. The first and corresponding authors were responsible for the final decision to submit the study for publication.

## Results

### Test enrolment

**Fig. 2:**
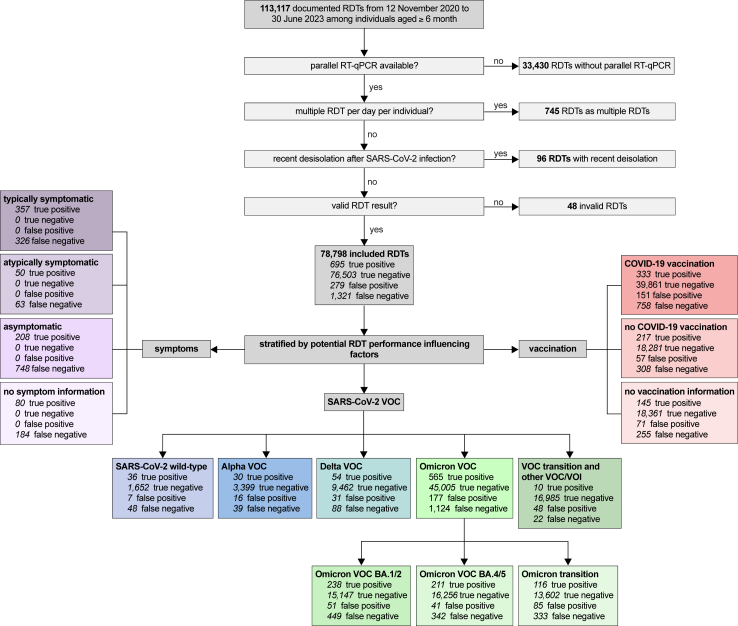
Enrolment of SARS-CoV-2 antigen rapid detection tests (RDTs), VOC: virus variant of concern, RDT: Antigen Rapid Detection Test, RT-qPCR: Quantitative reverse transcription-polymerase chain reaction.

Between 12 November 2020 and 30 June 2023, a total of 113,117 RDTs were performed and documented at the study centre from individuals aged ≥six month. After exclusion of RDTs without parallel RT-qPCR, multiple RDTs on one study day and in case of a recent de-isolation, and RDTs with invalid results, 78,798 paired RDT/RT-qPCR test results from 53,800 individuals could be included (Fig. 2).

Regarding the manufacturer 14.0% (11,021/78,798) of the paired RDTs were performed with NADAL®, 32.8% (25,882/78,798) with Panbio™ and 53.2% (41,895/78,798) with MEDsan® (Supplementary Figure S4b). The rate of invalid RDTs was comparable across the three products used: 0.045% (5/11,026) NADAL®, 0.058% (15/25,897) Panbio™ and 0.067% (28/41,923) MEDsan® (p = 0.70; Chi-Squared test).

### Study population

**Fig. 3:**
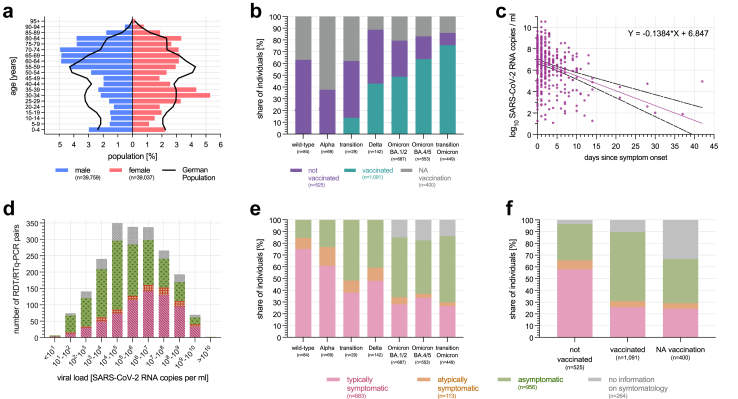
Characterisation of the study population. a) Age characterisation in categories of five years stratified by sex in reference to the German population (n = 78,798). b) COVID-19 vaccination status stratified by SARS-CoV-2 VOC among RT-qPCR positive samples (n = 2016). c) Viral load depending on days since symptom onset (n = 356). d) Distribution of viral load stratified by symptoms in absolute numbers among RT-qPCR positive samples (n = 2016). e) COVID-19 symptom status stratified by SARS-CoV-2 VOC among RT-qPCR positive samples (n = 2016). f) COVID-19 symptom status stratified by vaccination status among RT-qPCR positive samples (n = 2016). RDT: Antigen Rapid Detection Test. NA: no information available. Data source: Bayerisches Landesamt für Statistik, Robert Koch-Institut.^12^^,^^31^

The median age of the individuals included at paired RDT/RT-qPCR performance analysis was 54 years (range six month to 102 years, IQR: 31–70 years) covering 49.5% (39,037/78,798) female and 50.5% (39,759/78,798) male individuals (Fig. 3a). Two RDT/RT-qPCR test pairs were performed on individuals allocating themselves to diverse sex. The RDT/RT-qPCR test pairs included were performed in 87.3% (68,819/78,798) on patients, in 11.7% (9228/78,798) on accompanying individuals, and in 1.0% (751/78,798) on staff. Overall, a SARS-CoV-2 prevalence of 2.6% (2016/78,798) was detected with a median viral load of 5.6 (IQR: 4.1–7.2) log_10_ SARS-CoV-2 RNA copies/ml (Supplementary Figure S5).

Among positive RT-qPCR test results, 4.2% (84/2016) could be allocated to wild-type SARS-CoV-2, 3.4% (69/2016) to Alpha VOC, 7.0% (142/2016) to Delta VOC, 34.1% (687/2016) to Omicron BA.1/2 VOC, and 27.4% (553/2016) to Omicron BA.4/5 VOC. The remaining 23.4% (478/2016) could not be allocated to any VOC, but 93.9% (449/478) of those were in the transition phase in the Omicron VOC period (Fig. 2). Further 0.1% (2/2016) were molecularly allocated to the Iota VOI; for the single remaining sample (0.1%; 1/2016) no VOC could be finally allocated despite molecular testing.

Information on COVID-19 vaccination status was available among 76.1% (59,966/78,798) RDT-/RT-qPCR pairs: 31.5% (18,863/59,966) were conducted on unvaccinated, and 68.5% (41,103/59.966) on vaccinated individuals (Fig. 2). Among the remaining 23.9% (18,832/78,798) no information on COVID-19 vaccination status could be evaluated (Supplementary Figure S4). A progressive proportion of the included RDT/RT-qPCR pairs on vaccinated individuals were chronologically performed from the Delta VOC period onwards (Fig. 3b).

Among the RT-qPCR positive pairs, 33.9% (683/2016) presented with COVID-19 typical and 5.6% 113/2016) with atypical symptoms. 47.4% (956/2016) were asymptomatic (Fig. 2, Supplementary Figure S5). In 37.5% (356/686) of the typically symptomatic pairs, information on the number of days since symptom onset was available. The viral load decreased in the disease course (Fig. 3c).

### RDT performance compared to RT-qPCR: univariate analyses

**Fig. 4:**
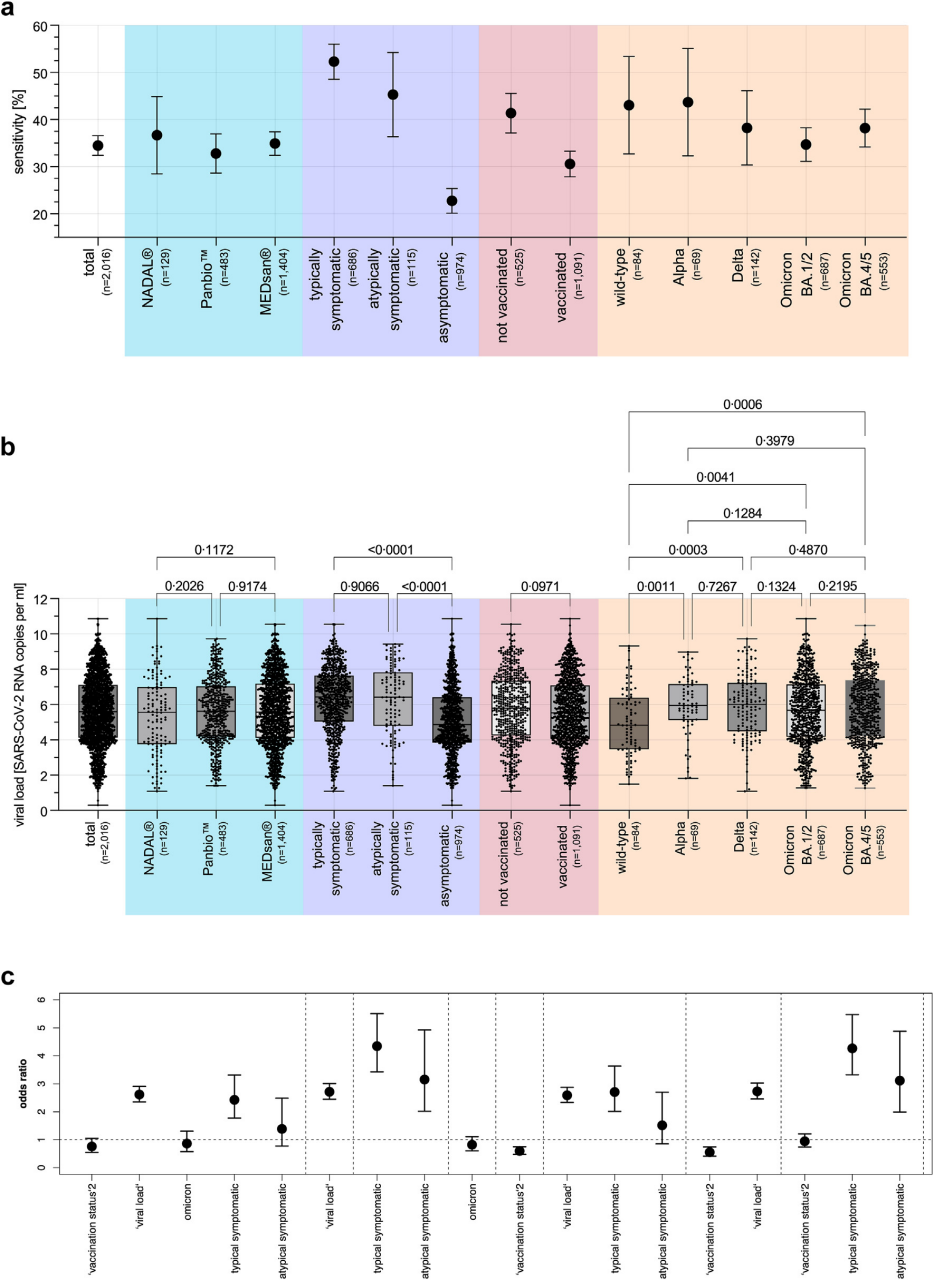
RDT sensitivity, SARS-CoV-2 viral load, and Odds Ratios of RDT performance influencing factors. a) RDT sensitivity overall and by RDT performance influencing factors (n = 2016). b) Viral load overall and by RDT performance influencing factors (n = 2016; Mann-Whitney-U tests). c) Odds Ratio of the several RDT performance influencing factors (n = 1472; linear regression models: full model including all influencing factors selected by the lasso step, single models for each influencing factor (viral load, symptoms, Omicron VOC, vaccination status), models including relevant combinations of influencing factors (viral load and symptoms, vaccination status and viral load, vaccination status and symptoms)). In the case of whiskers in the figures, these represent the respective 95% confidence intervals. RDT: Antigen Rapid Detection Test, VOC: virus variant of concern.

Overall, RDT sensitivity was 34.5% (695/2016; 95% CI 32.4–36.6%) and RDT specificity 99.6 (76,503/76,782; 95% CI 99.6–99.7%; Fig. 4a). No significant sensitivity differences could be observed between the manufacturers (all p > 0.40, Fisher's exact test; Fig. 4a).

Univariately, the sensitivity among asymptomatic individuals was significantly lower compared to typically and atypically symptomatic (both p < 0.0001, Fisher's exact test; Fig. 4a).

The sensitivity among vaccinated individuals was significantly lower compared to the unvaccinated (p < 0.0001, Fisher's exact test; Fig. 4a).

A trend in decreased sensitivity could be observed from the SARS-CoV-2 wild-type (42.9%; 36/84; 95% CI 32.8–53.5%) and Alpha VOC (42.3%; 30/69; 95% CI 32.4–55.2%) via Delta VOC (38.0%; 54/142; 95% CI 30.5–46.2%1124) to Omicron VOC (33.4%; 565/1689; 95% CI 31.2–35.7%) while it did not reach statistical significance (all p > 0.14, Fisher's exact test; Fig. 4a).

Regarding the viral load, no significant differences could be obtained comparing vaccinated to unvaccinated individuals (p = 0.10, Mann-Whitney-U test). The mean viral load of wild-type samples was significantly lower compared to Alpha VOC (p = 0.0011, Mann-Whitney-U test), Delta VOC (p = 0.0003, Mann-Whitney-U test), Omicron BA.1/2 VOC (p = 0.0041, Mann-Whitney-U test), and Omicron BA.4/5 VOC samples (p = 0.0006, Mann-Whitney-U test; Fig. 4b).

### Determinants of RDT performance

On the condition of data availability concerning vaccination status and VOC, 73.0% (1472/2016) of the SARS-CoV-2 positive test pairs could be considered for the employed lasso regression model.

Considering the full model only viral load and typical COVID-19 symptoms seem to influence RDT performance. Both factors significantly increased the odds of having a positive RDT result in case of a SARS-CoV-2 infection (p < 0.0001, logistic regression). In contrast, age (eliminated in the selection step), gender (p = 1.00, logistic regression), atypical symptoms (p = 0.99, logistic regression), vaccination status (p = 0.43, logistic regression), and Omicron VOC (p = 1.00, logistic regression) showed no direct influence (Fig. 4c, Supplementary Figure S6; Supplementary Statistics). However, to avoid misinterpretation, different models were considered to study the total effect of each individual factor as well as the total effect when several factors were combined in one model (Fig. 4c; Supplementary Statistics). As a result, the total effect of typical and atypical COVID-19 symptoms increases–around 80% for typical and over 120% for atypical symptoms–when estimated as a single factor or without accounting for viral load. Similarly, the odds ratio of vaccination status tends towards one when COVID-19 symptoms are excluded from the logistic regression model (Fig. 4c).

To assess for a potential bias due to including individuals more than once in the study, an additional analysis only including the first RDT/RT-qPCR of each individual was performed, confirming the results above (Supplementary Statistics).

To assess indirect effects, mediated by typical symptoms, a logistic regression model with typical COVID-19 symptoms as dependent variable showed that vaccination (OR: 0.35, 95% CI 0.26–0.46, p < 0.0001, logistic regression) and infections with Omicron VOC (OR: 0.54, 95% CI 0.39–0.76, p < 0.0001, logistic regression) significantly decreased the chance of presenting typical COVID-19 symptoms in case of an infection. Age (p = 0.08, logistic regression) and sex (p = 1.00, logistic regression) did not show a significant influence on typical symptoms (Supplementary Statistics).

## Discussion

Overall, in a cohort of 78,798 paired RDT/RT-qPCR test results, an RDT sensitivity of 34.5% was detected over the course of the COVID-19 pandemic. Our study found that RDTs were more sensitive in unvaccinated individuals compared to vaccinated individuals, a difference that was mediated by symptom status. Additionally, the decline in RDT sensitivity throughout the pandemic can primarily be attributed to the reduced prevalence of symptomatic infections among vaccinated individuals and individuals infected with Omicron VOC.

The sensitivity is at the lower end of the spectrum in terms of previous RDT performance analyses.^3^^,^^4^ The low sensitivity can be attributed to the study setting screening symptomatic and asymptomatic individuals in the clinical care setting.^3^^,^^4^^,^^9^ The chosen RDTs in the study performed moderately in such laboratory-based performance evaluations.^3^^,^^4^ It should also be noted that the RDTs used do not belong to the second generation of VOC-adapted RDTs, although based on the study results presented, the VOC does not directly influence the performance and therefore the first generation of RDTs can still be classified as equivalent.^32^

Our findings regarding the higher sensitivity of RDTs in unvaccinated individuals compared to vaccinated individuals align with previous data.^13^ In addition to previous studies including a preliminary analysis indicating a decrease in RDT sensitivity during the pandemic, our study was able to differentiate between the effects of vaccination and the effects of the VOC, both impairing sensitivity by reducing the occurrence of symptoms.^7^ Thanks to its longitudinal design and screening setting, our study demonstrates that this finding is mediated by the combined effects of vaccination and the Omicron VOC, leading to a reduced occurrence of typical symptoms in SARS-CoV-2 infections.^7^^,^^8^^,^^13^^,^^14^

The observation that RDTs perform worse in asymptomatic individuals has been reported in previous studies.^3^^,^^4^ This was either not interpreted or attributed to a lower viral load in asymptomatic individuals. However, an earlier regression analysis in a systematic review found a viral load-independent effect of symptoms, interpreting this cautiously as an indication for further studies, including standardized viral load determination.^4^ Our data—including this standardised viral load determination—clearly show that even among individuals with the same viral load (measured as the amount of amplifiable RNA), RDTs perform worse in asymptomatic individuals. This may be explained by a potentially higher nucleocapsid-to-RNA ratio in symptomatic individuals.^14^^,^^33^

The study's significant value becomes apparent as it systematically examined the RDT performance and its influencing factors in the clinical care reality over a long period of the COVID-19 pandemic, including the transition to endemicity. This includes factors that changed significantly during the pandemic–the respective dominant VOC and the COVID-19 vaccination status.^12^ The strengths of the study include its large sample size, structured data collection, robust infrastructure, and the qualifications of the personnel conducting the swabs. Studies of comparable size and questions only cover parts of the COVID-19 pandemic excluding consideration of Omicron BA.4/5 VOC.^9^^,^^15^ All swabs for RDT and RT-qPCR as well as the RDTs themselves were performed by trained staff from the university hospital with user support available at all times, minimising the influence of potential heterogeneities in sample collection, test execution, and interpretation to the best extent possible. In comparison to numerous published studies in the field of RDT performance analysis, the study represents a low prevalence of SARS-CoV-2 throughout the study period, with only 2.6% of included RDT/RT-qPCR test pairs showing a positive SARS-CoV-2 result.^3^^,^^4^ However, this reflects the chosen real study setting with RDT use for SARS-CoV-2 screening, including asymptomatic test subjects.

It is important to consider various limitations of the study when interpreting the data and drawing conclusions.

1. Due to the RDTs being used in immediate, point-of-care testing of patients and staff, the absolute numbers, and proportions of the RDT products used varied between different clinical departments and over the course of the study period. The assignment of individual RT-qPCR methods to each sample was random based on the capacities of virus diagnostics and the clinical urgency determined by the varying processing times of different RT-qPCR methods.
2. 32.6% of study participants provided samples for our study multiple times. To address this potential bias, additional analyses were conducted using only the first RDT/RT-qPCR pair for each individual, yielding comparable results, which can be found in the Supplementary Results.
3. The study participants included were only tested with one of the three selected RDTs. Laboratory analyses assessing the test performance of RDTs in an artificial setting may provide a more comprehensive to answer the issue of comparative performance analysis of different manufacturers, but they may not be as easily translated to the population's healthcare reality, especially with small sample sizes in the lab.
4. The molecular determination of the VOC using RT-qPCR was only carried out between January 2021 and January 2022. Therefore, a relevant proportion of RDT/RT-qPCR test pairs could only be allocated to especially wild-type SARS-CoV-2 (3.4%) and the Omicron VOC (83.9%) epidemiologically. Omicron VOC sublines could only be differentiated epidemiologically with a transitional period between and before sublines BA.1/2 and BA.4/5. Allocation to other Omicron VOC sublines was epidemiologically not possible, as no other subline group in Germany exceeded the defined 90% threshold epidemiologically during the study period, and especially towards the end of the study, multiple Omicron VOC sublines were present simultaneously due to the transition to endemicity.^11^^,^^12^
5. It should also be considered that vaccination data were only recorded for patients and accompanying individuals, and the recording of a COVID-19 vaccination started only from 23 May 2021. Thus, in the previous study period either no vaccination data were available, or the status “unvaccinated” could only be recorded based on age-stratified EMA approval data.^19^, ^20^, ^21^, ^22^

The observed reduction in sensitivity over the course of the pandemic can therefore be attributed to a decrease in the proportion of symptomatic infections due to vaccinations and the Omicron VOC, while the viral load has actually increased compared to the initial wild-type infections. Due to the reduced sensitivity of antigen rapid tests in asymptomatic individuals, their use in screening for future pandemic scenarios can only be considered if there is a significant improvement in sensitivity. Screening programs using antigen rapid tests in asymptomatic individuals, which were widely implemented in various countries during the pandemic and required substantial resources, must retrospectively be regarded as only conditionally useful. In contrast, antigen rapid tests provide a quick, widely available, and inexpensive testing method of acceptable performance for detecting SARS-CoV-2 infection in individuals with respiratory symptoms, regardless of VOC and vaccination status, even if the RT-qPCR remains the gold standard for SARS-CoV-2 diagnostics. Whether this also applies to other pathogens with potential pandemic threats, especially Influenza, needs to be investigated in further studies.

## Contributors

All authors had unlimited access to all data. Isabell Wagenhäuser, Alexander Gabel, and Manuel Krone take responsibility for the integrity of the data and the accuracy of the data analysis.

**Conceptualisation**: Isabell Wagenhäuser; Kerstin Knies; Ralf-Ingo Ernestus; Johannes Forster; Dirk Weismann; Benedikt Weißbrich; Johannes Liese; Christoph Härtel; Oliver Kurzai; Lars Dölken; Alexander Gabel; Manuel Krone.

**Methodology**: Isabell Wagenhäuser; Kerstin Knies; Alexander Gabel; Manuel Krone.

**Software**: Isabell Wagenhäuser; Alexander Gabel; Manuel Krone.

**Validation**: Isabell Wagenhäuser; Kerstin Knies; Tamara Pscheidl; Michael Eisenmann; Sven Flemming; Nils Petri; Miriam McDonogh; Agmal Scherzad; Daniel Zeller; Anja Gesierich; Anna Katharina Seitz, Regina Taurines; Ralf-Ingo Ernestus; Johannes Forster; Dirk Weismann; Benedikt Weißbrich; Johannes Liese; Christoph Härtel; Oliver Kurzai; Lars Dölken; Alexander Gabel; Manuel Krone.

**Formal analysis**: Isabell Wagenhäuser; Alexander Gabel; Manuel Krone.

**Investigation**: Isabell Wagenhäuser; Kerstin Knies; Tamara Pscheidl; Michael Eisenmann; Sven Flemming; Nils Petri; Miriam McDonogh; Agmal Scherzad; Daniel Zeller; Anja Gesierich; Anna Katharina Seitz; Regina Taurines.

**Resources**: Isabell Wagenhäuser; Kerstin Knies; Tamara Pscheidl; Michael Eisenmann; Sven Flemming; Nils Petri; Miriam McDonogh; Agmal Scherzad; Daniel Zeller; Anja Gesierich; Anna Katharina Seitz; Regina Taurines; Ralf-Ingo Ernestus; Dirk Weismann; Benedikt Weißbrich; Johannes Liese; Christoph Härtel; Lars Dölken.

**Data curation**: Isabell Wagenhäuser; Kerstin Knies; Tamara Pscheidl; Manuel Krone.

**Writing–Original Draft**: Isabell Wagenhäuser; Manuel Krone.

**Writing–Review & Editing**: Kerstin Knies; Tamara Pscheidl; Michael Eisenmann; Sven Flemming; Nils Petri; Miriam McDonogh; Agmal Scherzad; Daniel Zeller; Anja Gesierich; Anna Katharina Seitz; Regina Taurines; Ralf-Ingo Ernestus; Johannes Forster; Dirk Weismann; Benedikt Weißbrich; Johannes Liese; Christoph Härtel; Oliver Kurzai; Lars Dölken; Alexander Gabel.

**Visualisation**: Isabell Wagenhäuser; Alexander Gabel; Manuel Krone.

**Supervision**: Alexander Gabel; Manuel Krone.

**Project administration**: Isabell Wagenhäuser; Kerstin Knies; Alexander Gabel; Manuel Krone.

**Funding acquisition**: Oliver Kurzai; Lars Dölken.

The manuscript was reviewed and approved by all authors.

## Data sharing statement

Requests for access to an anonymised version of the complete dataset underlying this analysis after de-identification (text, tables, figures, and appendices) as well as the study protocol, statistical analysis plan, and analytic code is made available to researchers who provide a methodologically sound proposal to achieve aims in the approved proposal on request to the corresponding author.

The reproducible script of all statistical analyses can be accessed at https://github.com/AlexGa/SARS-CoV-2-Antigen-Rapid-Detection-Tests.

Additional data that underlies the results reported in this article, after de-identification (text, tables, figures, and appendices) as well as the study protocol, statistical analysis plan, and analytic code is made available to researchers who provide a methodologically sound proposal to achieve aims in the approved proposal on request to the corresponding author.

## Declaration of interests

Daniel Zeller receives honoraria from Angelini Pharma and Novartis outside of the submitted work. Manuel Krone receives honoraria from Abbott, GSK, Pfizer, and Sanofi outside of the submitted work. None of the other authors have any conflicts of interest to declare.

## Appendix A Supplementary data

Supplementary Methods, Figures and Tables

Supplementary Statistics
