# Supplementary material for: SARS-CoV-2 antigen rapid detection tests: test performance during the COVID-19 pandemic and the impact of COVID-19 vaccination
Source: eBioMedicine. 2024 Oct 10;109:105394. doi: 10.1016/j.ebiom.2024.105394 (PMC11663747; doi:10.1016/j.ebiom.2024.105394)
Supplement: Supplementary Methods, Figures and Tables [file mmc1.pdf]

Supplementary Material

Table of Contents

Supplementary Methods **1**

RDT implementation and deployment strategy throughout the course of the study *1*

Determination of COVID-19 vaccination status *1*

RT-qPCR analytical instruments *2*

Determination and allocation of SARS-CoV-2 virus variant of concern *2*

Supplementary Results **3**

Subgroup analysis of the first study participation of each subject *3*

Supplementary Tables and Figures **3**

Supplementary References **7**

Supplementary Methods

RDT implementation and deployment strategy throughout the course of the study

From 12 November 2020 to 24 November 2022, mandatory entry screening, i.e., RDT diagnostics upon admission, was conducted for patients and inpatient companions in all critically assessed areas of the hospital, such as emergency departments and delivery rooms, based on the prevailing evidence and pandemic situation. During periods of high COVID-19 incidence, from 1 February 2021, to 30 June 2021, and from 4 November 2021, to 24 November 2022, this mandatory entry screening was extended to all areas of the hospital, resulting in a universal RDT entry screening.(1, 2)

In addition, since 12 November 2020, employees with COVID-19 typical symptoms or contact with a SARS-CoV-2 positive person were examined using RDT/RT-qPCR test pairs at the central testing centre of the hospital. The documented RDT/RT-qPCR pairs were also included in the study.

From 25 November 2022, mandatory entry screening at the hospital, involving both RDT and RT-qPCR, was discontinued due to the general easing of the pandemic situation, and the sole use of RT-qPCR continued. The use of additional RDTs or RDTs without parallel RT-qPCR for screening was subsequently implemented and documented in risk-adapted, decentral concepts customised for their individual characteristics.

On 19 May 2023, with the complete transition from pandemic to endemic, this cross-clinic, individualised mandatory RDT deployment strategy and further diagnostic continuation were voluntary in individual clinics until 30 June 2023, which remarks the end of the study period (*Supplementary Figure 1*).(1-3)

Determination of COVID-19 vaccination status

The COVID-19 questionnaire was conducted as part of the standardised entry interview at the study centre from 31 May 2021 for the entire study period recording whether the patient or the accompanying person was immunised against COVID-19 with at least two doses of an EMA-approved COVID-19 vaccine or with at least one dose in addition to a confirmed PCR-confirmed SARS-CoV-2 infection. Until the most recent documentation of the vaccination status "unvaccinated", all this and previously conducted RDTs were classified as RDTs as "unvaccinated". From the first documentation of a vaccination onwards, all RDTs conducted for an individual were classified as conducted on a "vaccinated" subject. All RDTs performed before the age-stratified initial approval of a COVID-19 vaccine by the EMA were classified as "unvaccinated”. In the age group with a minimum age of 16 years, all RDTs before 21 December 2020 were classified as "unvaccinated”,(4) between 12 and 15 years all RDTs before 28 May 2021,(5) between five and 11 years all RDTs before 26 November 2021,(6) and between four years and six month all RDTs before 19 October 2022.(7)

RT-qPCR analytical instruments

The subsequent RT-qPCR analytical instruments were employed for the determination SARS-CoV-2 detection with determination of the viral load:

- (I) MagNaPure 96 / 7500 Real-Time PCR System / FTD SARS-CoV-2-PCR (target N/ORF1ab-gene, Roche Diagnostics, Rotkreuz, Switzerland / Thermo Fisher Scientific, Waltham MA, USA / Siemens Healthineers, Munich, Germany)
- (II) NeuMoDx™ (target N/Nsp2-gene, Qiagen, Hilden, Germany)
- (III) Alinity m (target RdRp/N-gene, Abbott Laboratories, Abbott Park IL, USA)
- (IV) QIAstat-Dx® (target RdRp/E-gene, Qiagen)
- (V) Xpert® Xpress SARS-CoV-2/Flu/RSV (target E/N2/RdRp-gene, Cepheid, Sunnyvale CA, USA)
- (VI) cobas® SARS-CoV-2 (target ORF1ab/E-gene, Roche Diagnostics)
- (VII) cobas® Liat (target ORF1a/b/N-Gen, Roche Diagnostics)
- (VIII) BIOFIRE® FILMARRAY® (target S-/M-gene, bioMérieux, Marcy-l'Étoile, France)

Since the two methods cobas® Liat and BIOFIRE® FILMARRAY® only enable qualitative SARS-CoV-2 RT-qPCR without quantification of a Cycle Threshold value (C_t-value), in the event of positive SARS-CoV-2 detection by one of these two methods, the RT-qPCR was repeated for quantification with Xpert® Xpress SARS-CoV-2/Flu/RSV in case of a positive cobas® Liat result and Xpert® Xpress SARS-CoV-2/Flu/RSV or NeuMoDx™ in case of a positive BIOFIRE® FILMARRAY® result.

Determination and allocation of SARS-CoV-2 virus variant of concern

Between 3 February 2021, and 19 January 2022, all RT-qPCR-positive samples with sufficient viral load underwent PCR with spike protein variant-specific primers to differentiate between current VOC using the VirSNiP SARS-CoV-2 Spike N501Y, del 69/70, E484K, N501Y, L452R, T478K, and 371L 373P 452R Kits (TIB molbiol, Berlin, Germany) carried out on a cobas z 480 analyzer (Roche Diagnostics, Rotkreuz, Switzerland). This method was chosen because VOC differentiation through multiple RT-qPCR tests showed reliable and high agreement with VOC determination using SARS-CoV-2 genome sequencing. In the case of combinations of results from this method that did not correspond to a common VOC, the described analytical procedure was carried out using SARS-CoV-2 Spike protein sequencing to molecularly determine SARS-CoV-2.(8) With very high COVID-19 incidence in the pandemic phase with almost complete dominance of the Omicron VOC at very high RT-qPCR sample volume, the laboratory analytical VOC determination was finally terminated on 19 January 19 2022.(1)

For all RT-qPCR samples collected outside the period of molecular VOC determination, the VOC was epidemiologically assigned to the RT-qPCR sample. For RT-qPCR samples collected within the period of laboratory VOC determination but with low viral load making molecular VOC determination not possible or in VOC determination, no VOC could be clearly diagnosed; if available, the VOC was derived from the known SARS-CoV-2 infection source; otherwise, the VOC was similarly epidemiologically assigned. This was done according to the following principle: All RT-qPCR samples included before 3 February 2021, were assumed to be SARS-CoV-2 wild-type since, chronologically according to epidemiology, the first VOC detections in Germany occurred after this date. Epidemiologically, the RT-qPCR sample was always assigned to the VOC responsible for more than 90% of SARS-CoV-2 cases in Germany in the calendar week of sample collection. If no VOC in a calendar week caused more than 90% of SARS-CoV-2 cases, no VOC assignment to a RT-qPCR positive sample was possible; those intervals were referred to as the "transition period".(2)

With the spread of the Omicron VOC, there was also the epidemiological peculiarity that various Omicron VOC sublines occurred during the entire Omicron VOC-caused phase of the COVID-19 pandemic, with the associated transition to the endemic phase. From the third calendar week of 2022 until the end of the study, epidemiologically, in total, at least 90% of SARS-CoV-2 cases were caused by different Omicron VOC sublines. Since only for the two Omicron VOC subline cohorts Omicron BA.1-2 VOC and Omicron BA.4-5 VOC, a clear VOC assignment could be defined by reaching the defined epidemiological threshold of 90% during the respective periods, outside of this period from the third calendar week of 2022, an "Omicron VOC transition period" was defined where parallel different sublines were detectable, making up more than 90% of SARS-CoV-2 cases, but no single VOC or VOI reached this threshold. If in the molecular analysis, the VOC was only differentiated as Omicron VOC, in the case of an analysis stratified according to the Omicron VOC sublines BA.1-2 and BA.4-5, the epidemiological allocation of the molecular result was preferred as an exception (*Supplementary Table 1*).(2)

Supplementary Statistics

The reproducible script of all statistical analyses can be accessed at https://github.com/AlexGa/SARS-CoV-2-Antigen-Rapid-Detection-Tests.

Supplementary Results

Subgroup analysis of the first study participation of each subject

Considering only the first participation of the included individuals 53,800 RDT/RT-qPCR pairs remained for the subgroup analysis including 26,801 (49·8%) female and 26,997 (50·2%) male individuals. Two individuals allocated themselves to diverse gender. The median age was 53 (IQR: 30-70) years. For the first study participation, 8,128 (15·1%) RDTs were performed using the Nadal® RDT, 17,630 (32·8%) using the Panbio™ RDT and 28,042 (52·1%) using the MEDsan® RDT. The subcohort consisted of 47,313 (87·9%) patients, 5,922 (11·0%) accompanying persons and 565 (1·1%) employees.

The RDT sensitivity was 37·2% (528/1,420; 95% CI 34·7%-39·7%), the specificity 99·6% (52,184/52,380; 95% CI 99·6%-99·7%), the Positive Predictive Value 72·9% (528/724; 95% CI 69·6%-76·0%) and the Negative Predictive Value 98·3 (52,184/53,075; 95% CI 98·2%-98·4%).

Supplementary Tables and Figures

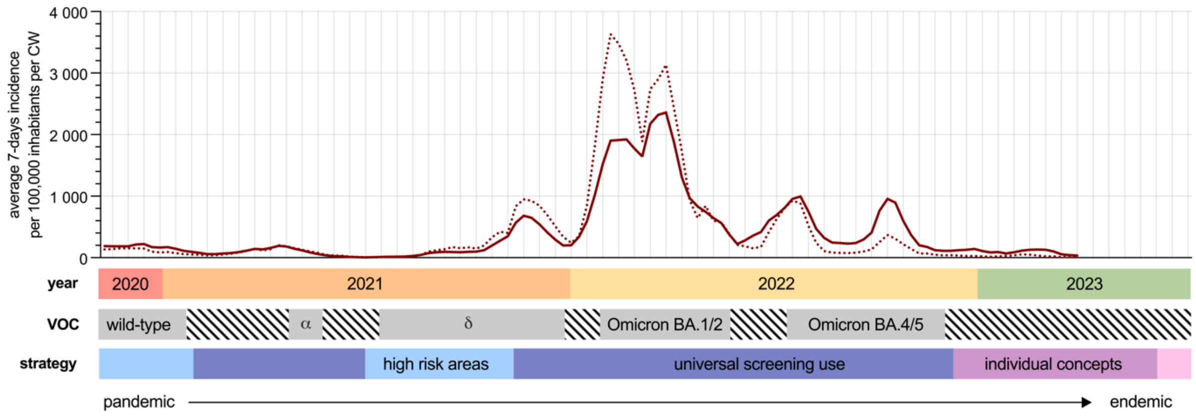

Supplementary Figure 1: SARS-CoV-2 incidence in Bavaria during the COVID-19 pandemic, the SARS-CoV-2 VOCs and the RDT deployment strategy of the study

The solid curve shows the incidence in the general population, the dashed curve the incidence among children and adolescents aged 0-15 years. The data was not available from the provider until the end of the study.

VOC: virus variant of concern

Data source: Robert Koch-Institut, Bayerisches Landesamt für Gesundheit und Lebensmittelsicherheit(1, 2)

calendar week (CW)	epidemiological VOC-allocation
CW 46/2020 - CW 3/2021	SARS-CoV-2 wild-type
CW 4/2021 - CW 15/2021	*VOC transition*
CW 16/2021 - CW 17/2021	Alpha VOC
CW 18/2021 - CW 19/2021	*VOC transition*
CW 20/2021 - CW 21/2021	Alpha VOC
CW 22/2021 - CW 27/2021	*VOC transition*
CW 28/2021 - CW 50/2021	Delta VOC
CW 51/2021 - CW 2/2022	*VOC transition*
CW 3/2022 - CW 20/2022	Omicron BA.1-2 VOC
CW 21/2022 - CW 26/2022	*Omicron VOC transition*
CW 27/2022 - CW 47/2022	Omicron BA.4-5 VOC
CW 48/2022 - CW 26/2023	*Omicron VOC transition*

Supplementary Table 1: Epidemiological VOC assignment by calendar week (CW) in the study period

VOC: virus variant of concern

Data source: Robert Koch-Institut(2)

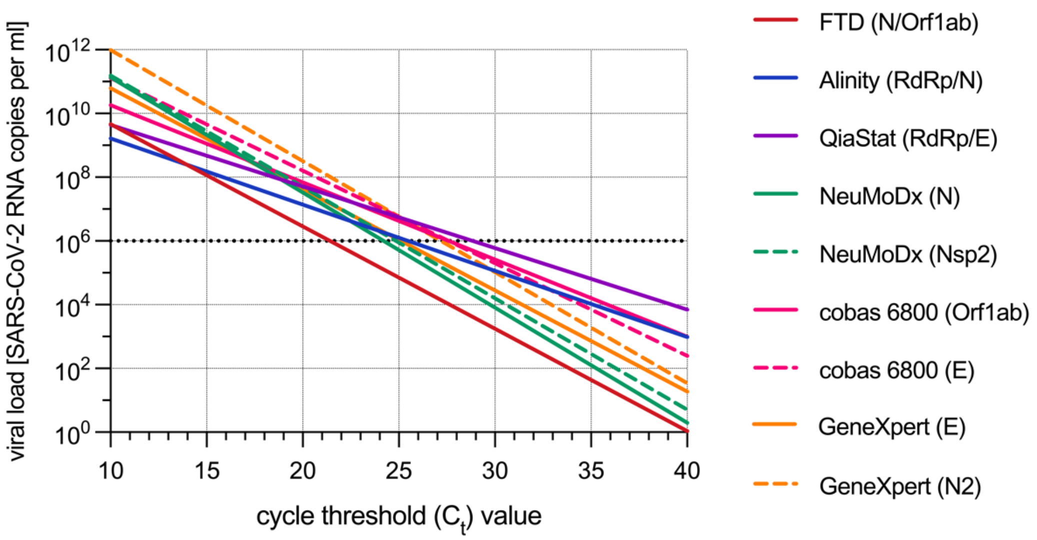

Supplementary Figure 2: Viral load as a function of the C_t value stratified according to the RT-qPCR method

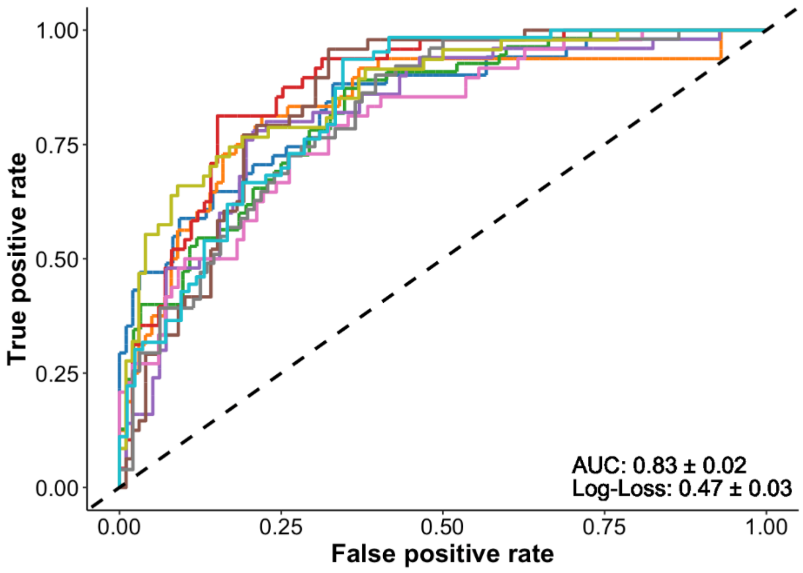

Supplementary Figure 3: Lasso regression model: AUC (area under the curve) of the ROC (receiver operating characteristic) curve (n=1,472)

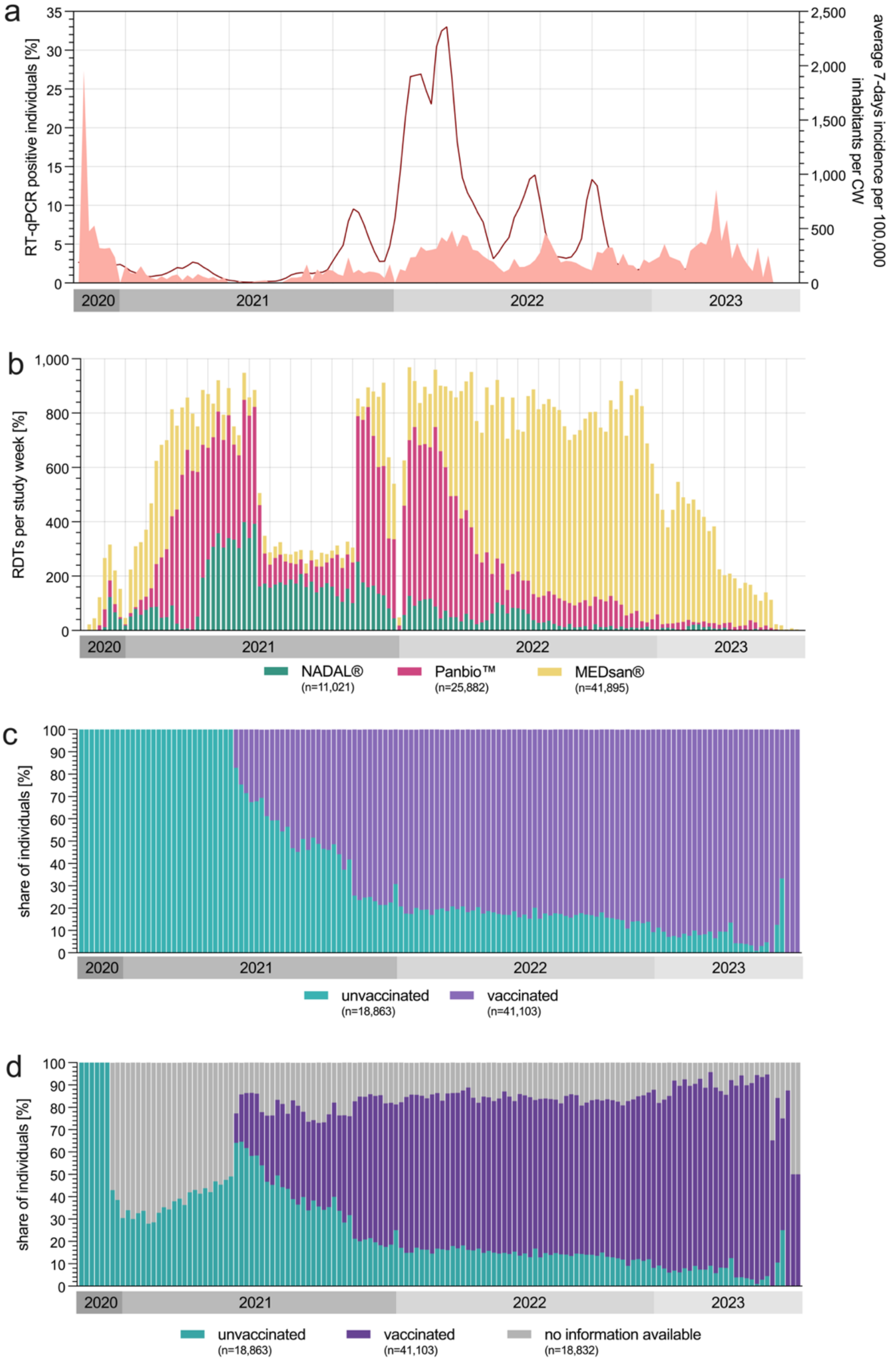

Supplementary Figure 4: SARS-CoV-2 prevalence, number of tests, and COVID-19 vaccination status over the course of the study

4a) Fraction of RT-qPCR positive RDT/RT-qPCR test pairs per study week (bright red filled curve) and SARS-CoV-2 incidence per 100,000 inhabitants per CW in the study region (red line)

4b) Number of RDTs per CW stratified by RDT manufacturer

4c) Fraction of COVID-19 vaccination status of RDT/RT-qPCR test pairs per study week

4d) Fraction of COVID-19 vaccination status of RDT/RT-qPCR test pairs per study week including test pairs without available vaccination status

CW: calendar week

Data source: Landesamt für Gesundheit und Lebensmittelsicherheit(1), European Medicines Agency (EMA)(4-7)

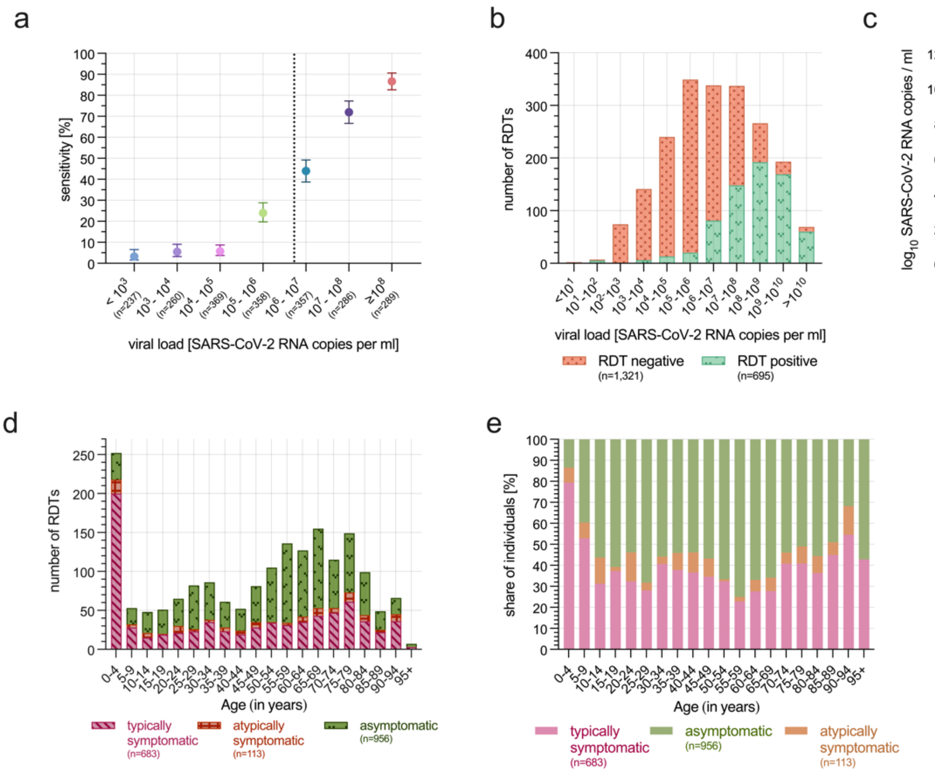

Supplementary Figure 5: Distribution of potential RDT performance influencing factors
5a) RDT sensitivity depending on viral load in categories (n=2,016)
5b) RDT test result depending on viral load in absolute numbers (n=2,016)
5c) Viral load stratified by RDT test result (n=2,016; Mann-Whitney-U test)
5d) Age distribution of RDTs stratified by COVID-19 symptomatology (n=1,752)
5e) Proportionate distribution of symptomatology stratified by age category (n=1,752)
RDT: Antigen Rapid Detection Test

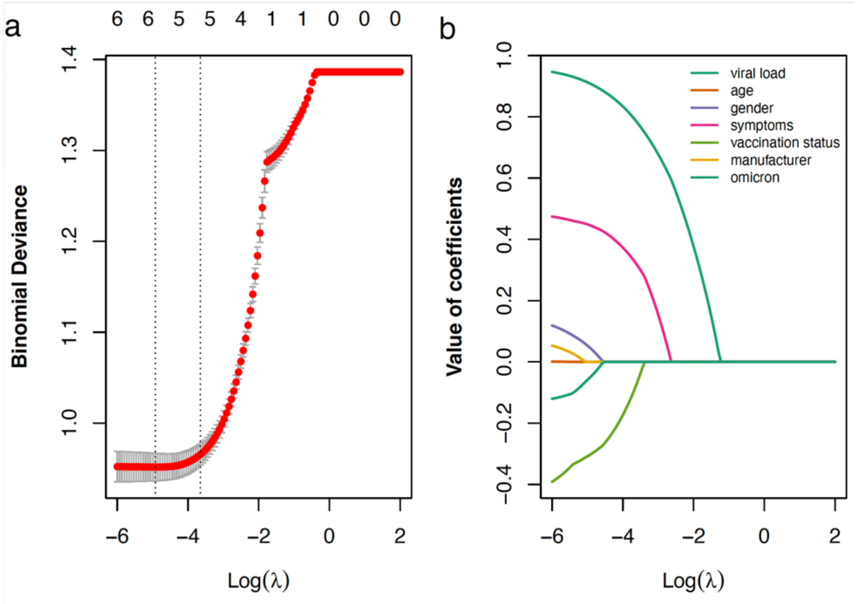

Supplementary Figure 6: Lasso regression for detection of influencing factors associated to RDT sensitivity
6a) Tenfold cross-validation procedure to determine optimal lambda parameter based on minimal mean-squared error (n=1,427)
6b) Illustrating the shrinkage of coefficients (factors) towards zero with increasing lambda values (n=1,427)

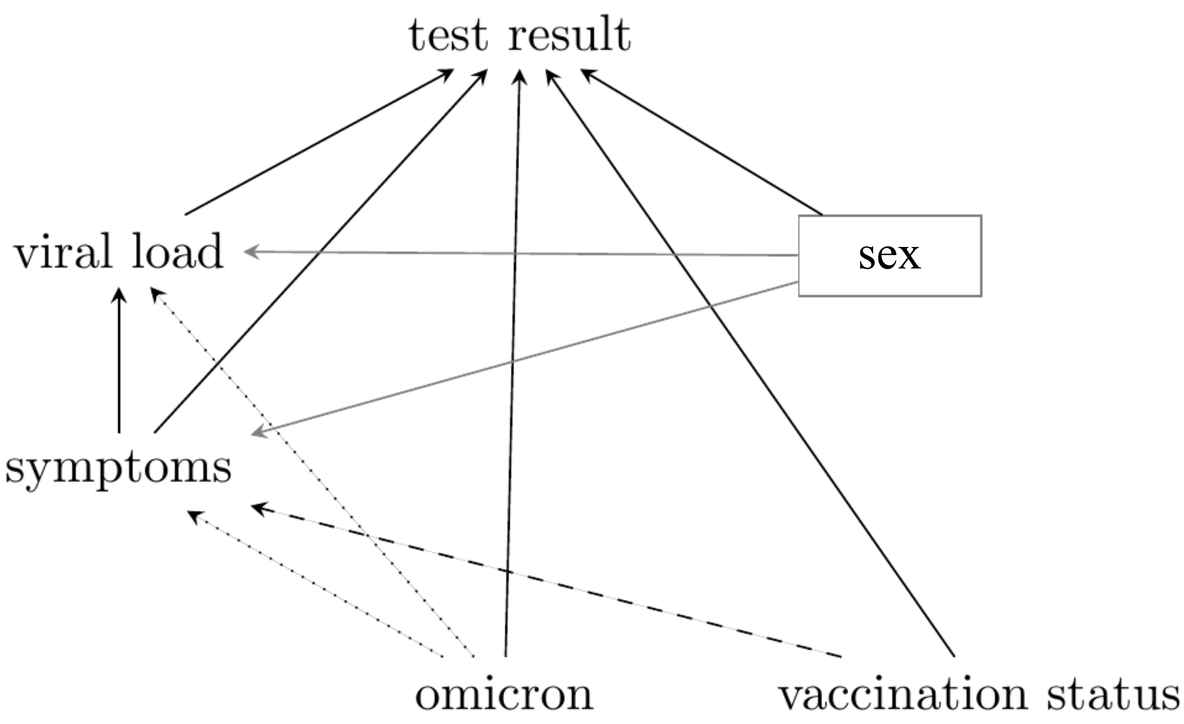

Supplementary Figure 7: Plausible causal diagram for the effects on RDT test sensitivity based on the results of different regression models

Supplementary References

1. Bayerisches Landesamt für Gesundheit und Lebensmittelsicherheit: Übersicht der Fallzahlen von Coronavirusinfektionen in Bayern 2023 [Available from: https://www.lgl.bayern.de/gesundheit/infektionsschutz/infektionskrankheiten_a_z/coronavirus/karte_coronavirus/ (Accessed 31 December 2023).
2. Robert Koch-Institut (RKI): Anzahl und Anteile von VOC und VOI in Deutschland 2023 [Available from: https://www.rki.de/DE/Content/InfAZ/N/Neuartiges_Coronavirus/Daten/VOC_VOI_Tabelle.html (Accessed 23 October 2023).
3. Brust KB, Kobayashi T, Diekema DJ. Asymptomatic Testing of Hospital Admissions for SARS-CoV-2: Is it OK to Stop? Clinical Infectious Diseases. 2023;78(2):356-60.
4. European Medicines Agency (EMA): EMA recommends first COVID-19 vaccine for authorisation in the EU 2020 [Available from: https://www.ema.europa.eu/en/news/ema-recommends-first-covid-19-vaccine-authorisation-eu (Accessed 19 January 2024).
5. European Medicines Agency (EMA): First COVID-19 vaccine approved for children aged 12 to 15 in EU 2021 [Available from: https://www.ema.europa.eu/en/news/first-covid-19-vaccine-approved-children-aged-12-15-eu (Accessed 20 January 2024).
6. European Medicines Agency (EMA): Comirnaty COVID-19 vaccine: EMA recommends approval for children aged 5 to 11 2021 [Available from: https://www.ema.europa.eu/en/news/comirnaty-covid-19-vaccine-ema-recommends-approval-children-aged-5-11 (Accessed 20 January 2024).
7. European Medicines Agency (EMA): EMA recommends approval of Comirnaty and Spikevax COVID-19 vaccines for children from 6 months of age 2022 [Available from: https://www.ema.europa.eu/en/news/ema-recommends-approval-comirnaty-and-spikevax-covid-19-vaccines-children-6-months-age (Accessed 20 January 2024).
8. Lind A, Barlinn R, Landaas ET, Andresen LL, Jakobsen K, Fladeby C, et al. Rapid SARS-CoV-2 variant monitoring using PCR confirmed by whole genome sequencing in a high-volume diagnostic laboratory. Journal of Clinical Virology. 2021;141:104906.
