# Supplementary material for: SARS-CoV-2 antigen rapid detection tests: test performance during the COVID-19 pandemic and the impact of COVID-19 vaccination
Source: eBioMedicine. 2024 Oct 10;109:105394. doi: 10.1016/j.ebiom.2024.105394 (PMC11663747; doi:10.1016/j.ebiom.2024.105394)
Supplement: Supplementary Statistics [file mmc2.zip › Supplementary_Statistical_Analysis.html]

SARS-CoV-2 Antigen Rapid Detection Tests: test performance during the COVID-19 pandemic and the impact of COVID-19 vaccination


Code 

- Show All Code
- Hide All Code

# SARS-CoV-2 Antigen Rapid Detection Tests: test performance during the COVID-19 pandemic and the impact of COVID-19 vaccination

Isabell Wagenhäuser1,2; Kerstin Knies3; Tamara
Pscheidl1,4; Michael Eisenmann1; Sven
Flemming5; Nils Petri2; Miriam
McDonogh4,6; Agmal Scherzad7; Daniel
Zeller8; Anja Gesierich9; Anna Katharina
Seitz10; Regina Taurines11; Ralf-Ingo
Ernestus12; Johannes Forster13; Dirk
Weismann2; Benedikt Weißbrich3; Johannes
Liese14; Christoph Härtel14; Oliver
Kurzai13,15; Lars Dölken3; Alexander
Gabel1,16; Manuel Krone1,13

1 Infection Control and Antimicrobial Stewardship Unit,
University Hospital Würzburg, Josef-Schneider-Str. 2, 97080 Würzburg,
Germany

2 Department of Internal Medicine I, University Hospital
Würzburg, Oberdürrbacher Str. 6, 97080 Würzburg, Germany

3 Institute for Virology and Immunobiology,
Julius-Maximilians-Universität Würzburg, Versbacher Str. 7, 97078
Würzburg, Germany

4 Department of Anaesthesia and Critical Care, University
Hospital Würzburg, Oberdürrbacher Str. 6, 97080 Würzburg, Germany

5 Department of General, Visceral, Transplantation,
Vascular, and Paediatric Surgery, University Hospital Würzburg,
Oberdürrbacher Str. 6, 97080 Würzburg, Germany

6 Department of Orthopaedic Trauma, Hand, Plastic, and
Reconstructive Surgery, University Hospital Würzburg, Oberdürrbacher
Str. 6, 97080 Würzburg, Germany

7 Department of Otorhinolaryngology, Plastic, Aesthetic,
and Reconstructive Head and Neck Surgery, University Hospital Würzburg,
Josef-Schneider-Str. 11, 97080 Würzburg, Germany

8 Department of Neurology, University Hospital Würzburg,
Josef-Schneider-Str. 11, 97080 Würzburg, Germany

9 Department of Dermatology, Venerology, and Allergology,
University Hospital Würzburg, Josef-Schneider-Str. 2, 97080 Würzburg,
Germany

10 Department of Urology, University Hospital Würzburg,
Oberdürrbacher Str. 6, 97080 Würzburg, Germany

11 Department of Child and Adolescent Psychiatry,
Psychosomatics, and Psychotherapy, University Hospital Würzburg,
Margarete-Höppel-Platz 1, 97080 Würzburg

12 Department of Neurosurgery, University Hospital
Würzburg, Josef-Schneider-Str. 11, 97080 Würzburg, Germany

13 Institute for Hygiene and Microbiology,
Julius-Maximilians-Universität Würzburg, Josef-Schneider-Str. 2, 97080
Würzburg, Germany

14 Department of Paediatrics, University Hospital
Würzburg, Josef-Schneider-Str. 2, 97080 Würzburg, Germany

15 Leibniz Institute for Natural Product Research and
Infection Biology – Hans-Knoell-Institute, Beutenbergstraße 13, 07745
Jena, Germany

16 Helmholtz Institute for RNA-based Infection Research
(HIRI), Helmholtz Centre for Infection Research (HZI),
Josef-Schneider-Str. 2, 97080 Würzburg, Germany

**Corresponding author:** Manuel Krone, Infection
Control and Antimicrobial Stewardship Unit, University Hospital
Würzburg

# Libraries and Functions

```
library(dplyr)
library(writexl)
library(tidyr)
library(pROC)
library(ggplot2)
library(kableExtra)
library(knitr)
source("scripts/helper_functions.R")
```

```
setwd("~/CoVacSer/RDT_4/Manuscript/git/SARS-CoV-2-Antigen-Rapid-Detection-Tests/")

plt_dir <- file.path("plots/lasso_test_results")
res_dir <- file.path("results/lasso_test_results")

if(!dir.exists(plt_dir)){
  dir.create(plt_dir)
}

if(!dir.exists(res_dir)){
  dir.create(res_dir)
}

df <- readr::read_csv2("data/data.csv") %>% 
      mutate(manufacturer = as.factor(manufacturer)) %>% 
      dplyr::rename(sex = gender)
```

# Factors influencing test result of RDT

**ASSUMPTION OF THE ABSENCE OF MULTICOLLINEARITY**

```
df_box_tid <- df %>% dplyr::mutate(symptoms1 = if_else(symptoms == 1, true = 1, false = 0),
                           symptoms2 = if_else(symptoms == 2, true = 1, false = 0),
                           omicron = as.numeric(omicron),
                           `vaccination status` = as.numeric(`vaccination status`),
                           `viral load` = as.numeric( `viral load`),
                           sexW = if_else(sex == "W", true = 1, false = 0),
                           manufacturer1 = if_else(manufacturer == 1, true = 1, false = 0),
                           manufacturer2 = if_else(manufacturer == 2, true = 1, false = 0)) %>%
                dplyr::select(-symptoms, -manufacturer, -sex)

png(filename = file.path(plt_dir, paste0("Scatter_all_pairs.png")),units="px", width=5000, height=5000, res=300)
pairs(df_box_tid %>% dplyr::select(-ID, -`test result`), lower.panel = panel.cor, pch = 20)
dev.off()

pairs(df_box_tid %>% dplyr::select(-ID, -`test result`), lower.panel = panel.cor, pch = 20)
```

**ASSUMPTION OF LINEARITY OF INDEPENDENT VARIABLES AND LOG
ODDS**

Check with Box-Tidwell test with transformed continuous variables

```
lreg <- glm(`test result` ~ age + ageTrans + `viral load` + viral_loadTrans + sexW + symptoms1 + symptoms2 + 
              `vaccination status` + omicron + manufacturer1 + manufacturer2, 
            data = df_box_tid %>% mutate(ageTrans = age * log(age),
                                 viral_loadTrans = `viral load` * log(`viral load`)), 
            family=binomial(link="logit"))

as.data.frame(summary(lreg)$coefficients) %>% 
  as_tibble(rownames = "Feature") %>% 
  mutate(across(where(is.double), 
                ~ format(.x, digits = 2, scientific = T))) %>% 
  knitr::kable()
```

| Feature | Estimate | Std. Error | z value | Pr(>|z|) |
| --- | --- | --- | --- | --- |
| (Intercept) | -4.5e+00 | 1.8e+00 | -2.5e+00 | 1.4e-02 |
| age | -7.4e-03 | 4.1e-02 | -1.8e-01 | 8.6e-01 |
| ageTrans | 2.1e-03 | 8.6e-03 | 2.4e-01 | 8.1e-01 |
| `viral load` | 2.3e-02 | 8.4e-01 | 2.7e-02 | 9.8e-01 |
| viral\_loadTrans | 3.3e-01 | 3.0e-01 | 1.1e+00 | 2.7e-01 |
| sexW | 1.7e-01 | 1.5e-01 | 1.1e+00 | 2.7e-01 |
| symptoms1 | 8.8e-01 | 1.7e-01 | 5.3e+00 | 1.0e-07 |
| symptoms2 | 3.2e-01 | 3.1e-01 | 1.0e+00 | 3.0e-01 |
| `vaccination status` | -3.5e-01 | 2.1e-01 | -1.6e+00 | 1.0e-01 |
| omicron | -1.2e-01 | 2.3e-01 | -5.2e-01 | 6.0e-01 |
| manufacturer1 | 2.2e-02 | 3.4e-01 | 6.4e-02 | 9.5e-01 |
| manufacturer2 | 7.8e-02 | 3.1e-01 | 2.5e-01 | 8.0e-01 |

## Adult data set with all variants

```
y <- df %>% pull(`test result`)
X <- df %>% dplyr::select(-ID, -`test result`) %>%
            dplyr::mutate(symptoms = factor(symptoms),# + 1, levels = 1:3),
                          sex = factor(sex, levels = c("M", "W")),
                          `vaccination status` = as.factor(as.numeric(`vaccination status`)),
                          manufacturer = factor(manufacturer))
X_lasso <- X %>% mutate(across(where(is.factor), .fns = as.integer)) %>% as.matrix()

## 3. 10-fold cross validation to estimate lambda

set.seed(10)
lambdas2try <- exp(seq(-6, 2, length.out = 120))
lasso_cv <- glmnet::cv.glmnet(x = X_lasso, y = y, family = "binomial", alpha = 1, nfolds = 10, lambda = lambdas2try,
                              intercept = FALSE, standardize = TRUE)


model_all_lambdas <- glmnet::glmnet(x = X_lasso, y = y, family = "binomial", alpha = 1, nfolds = 10, lambda = lambdas2try)
par(mfcol = c(1,2), mar=c(5,4.,2.,1)+0.1, font.lab = 2)
plot(lasso_cv)
put.fig.letter(label="a", location="topleft", font=1, cex = 1.5)
matplot(log(model_all_lambdas$lambda), t(as.matrix(model_all_lambdas$beta)), type = "l", lty = rep(c(1,2), each = 9), col = RColorBrewer::brewer.pal(6,"Dark2"), lwd = 2,
        xlab = parse(text = ("Log(lambda)")), ylab = "Value of coefficients")
legend("topright", lty = rep(c(1,2), each = 9), col = rep(RColorBrewer::brewer.pal(6,"Dark2"), 2) , lwd = 1.5, cex = .7,
       legend = rownames(model_all_lambdas$beta), bty = "n")
put.fig.letter(label="b", location="topleft", font=1, cex = 1.5)
```

```
pdf(file.path(plt_dir, "LASSO_REGRESSION.pdf"), height = 5)
par(mfcol = c(1,2), mar=c(5,4.,2.,1)+0.1, font.lab = 2)
plot(lasso_cv)
put.fig.letter(label="a", location="topleft", font=1, cex = 1.5)
matplot(log(model_all_lambdas$lambda), t(as.matrix(model_all_lambdas$beta)), type = "l", lty = rep(c(1,2), each = 9), col = RColorBrewer::brewer.pal(6,"Dark2"), lwd = 2,
        xlab = parse(text = ("Log(lambda)")), ylab = "Value of coefficients")
legend("topright", lty = rep(c(1,2), each = 9), col = rep(RColorBrewer::brewer.pal(6,"Dark2"), 2) , lwd = 1.5, cex = .7,
       legend = rownames(model_all_lambdas$beta), bty = "n")
put.fig.letter(label="b", location="topleft", font=1, cex = 1.5)
dev.off()
```

## Perform lasso for minimal lambda

```
model_all_lambdas <- glmnet::glmnet(x = X_lasso, y = y, family = "binomial", alpha = 1, nfolds = 10, lambda = lasso_cv$lambda.min)

lambda_cv <- lasso_cv$lambda.min
lasso_best <- broom::tidy(lasso_cv)[lasso_cv$index,] %>% dplyr::rename(MSE = estimate)
lasso_best %>% knitr::kable(digits = 3)
```

| lambda | MSE | std.error | conf.low | conf.high | nzero |
| --- | --- | --- | --- | --- | --- |
| 0.007 | 0.952 | 0.015 | 0.936 | 0.967 | 6 |
| 0.026 | 0.965 | 0.012 | 0.954 | 0.977 | 5 |

## ROC lasso regression

## Final logistic regression based on selected features

```
## Extract varaibles unequal zero after lasso
lasso_vars_gt_zero <- rownames(model_all_lambdas$beta)[as.matrix(model_all_lambdas$beta)[,1] != 0]
```

viral load, sex, symptoms, vaccination status, omicron

DAG: Associated features

## Logistic regression including all factors (Model 8)

```
df_logit <- df %>% dplyr::select(`test result`, matches(paste0(lasso_vars_gt_zero, collapse = "|"))) %>%
            dplyr::mutate(symptoms = factor(symptoms),
                          sex = factor(sex, levels = c("M", "W")),
                          `vaccination status` = as.factor(as.numeric(`vaccination status`)))

# Perform final logistic regression with shrinked data set
glm_full <- glm(`test result` ~ .,data = df_logit, family=binomial(link='logit'))

df_coefs <- as.data.frame(summary(glm_full)$coefficients) %>% as_tibble(rownames = "Feature")

odds_ratio <- data.frame(odds_ratio = exp(coefficients(glm_full)[-1])) %>% as_tibble(rownames = "Feature")
odds_ratio_ci <- data.frame(exp(confint.default(glm_full)[-1,]), check.names = F) %>% as_tibble(rownames = "Feature")

# Store odds ratios and p values of lasso regression

odds_ratio %>% inner_join(odds_ratio_ci) %>% inner_join(df_coefs) %>%
  writexl::write_xlsx(path = file.path(res_dir, "lasso_coefs_odds_ratios.xlsx"))

odds_ratio_ci <- odds_ratio_ci %>% as.data.frame()

# Plot odds ratios and CI

pdf(file.path(plt_dir, "odds_ratio.pdf"), width = 20, height = 7)
par(mgp = c(2.5,1,0), font.lab = 2, mfrow=c(1,1), mar = c(4.1, 4.1, 0.2, 0.2))
plot(seq_len(nrow(odds_ratio)), odds_ratio %>% pull(odds_ratio),
     ylim = c(-1,6), pch = 20, cex = 3, xaxt = "n", xlab = "coefficients",  ylab = "odds ratio")
sapply(seq_len(nrow(odds_ratio)), function(i){
  arrows(x0=i, y0=odds_ratio_ci[i,2], x1=i, y1=odds_ratio_ci[i,3], code=3, col="black", lwd=2, angle=90, length=0.25)
})
axis(side = 1, at = seq_len(nrow(odds_ratio)), labels = odds_ratio %>% pull(Feature))
abline(h = 1, lty = 2)
dev.off()

# Write data into excel

sample_sizes_df <- df_logit %>% 
                    dplyr::select(matches(paste0(lasso_vars_gt_zero[!lasso_vars_gt_zero %in% c("age", "viral load")], collapse = "|"))) %>% 
                    mutate(omicron = factor(omicron)) %>%
  tidyr::pivot_longer(cols = matches("*"), names_to = "feature", values_to = "value", values_transform = list(value = as.character)) %>%
  group_by(feature, value) %>% count() %>%
  group_by(feature) %>%
  mutate(rel = n/sum(n))
  
writexl::write_xlsx(sample_sizes_df, path = file.path(res_dir, "sample_sizes_for_features.xlsx"))

par(mgp = c(2.5,1,0), font.lab = 2, mfrow=c(1,1), mar = c(4.1, 4.1, 0.2, 0.2))
plot(seq_len(nrow(odds_ratio)), odds_ratio %>% pull(odds_ratio),
     ylim = c(-1,6), pch = 20, cex = 3, xaxt = "n", xlab = "coefficients",  ylab = "odds ratio")
sapply(seq_len(nrow(odds_ratio)), function(i){
  arrows(x0=i, y0=odds_ratio_ci[i,2], x1=i, y1=odds_ratio_ci[i,3], code=3, col="black", lwd=2, angle=90, length=0.25)
})
axis(side = 1, at = seq_len(nrow(odds_ratio)), labels = odds_ratio %>% pull(Feature))
abline(h = 1, lty = 2)
```

**Odds ratio table**

```
padj_df <- data.frame(Feature = df_coefs$Feature, 
                      p.adj = p.adjust(df_coefs$`Pr(>|z|)`, method = "BY"))

sum_odds_ratio_table <- odds_ratio %>% 
                        inner_join(odds_ratio_ci) %>% 
                        inner_join(df_coefs) %>%
                        inner_join(padj_df)

sum_odds_ratio_table %>% 
  mutate(across(where(is.double), 
                ~ format(.x, digits = 3, scientific = TRUE))) %>% 
  knitr::kable()
```

| Feature | odds\_ratio | 2.5 % | 97.5 % | Estimate | Std. Error | z value | Pr(>|z|) | p.adj |
| --- | --- | --- | --- | --- | --- | --- | --- | --- |
| `viral load` | 2.62e+00 | 2.35e+00 | 2.91e+00 | 9.61e-01 | 5.40e-02 | 1.78e+01 | 8.61e-71 | 1.56e-69 |
| sexW | 1.15e+00 | 8.65e-01 | 1.53e+00 | 1.40e-01 | 1.45e-01 | 9.62e-01 | 3.36e-01 | 1.00e+00 |
| symptoms1 | 2.42e+00 | 1.77e+00 | 3.31e+00 | 8.86e-01 | 1.59e-01 | 5.55e+00 | 2.80e-08 | 1.70e-07 |
| symptoms2 | 1.39e+00 | 7.73e-01 | 2.48e+00 | 3.26e-01 | 2.98e-01 | 1.09e+00 | 2.74e-01 | 9.94e-01 |
| `vaccination status`2 | 7.58e-01 | 5.47e-01 | 1.05e+00 | -2.77e-01 | 1.66e-01 | -1.67e+00 | 9.57e-02 | 4.34e-01 |
| omicron | 8.62e-01 | 5.71e-01 | 1.30e+00 | -1.48e-01 | 2.10e-01 | -7.04e-01 | 4.81e-01 | 1.00e+00 |

### Group sizes

```
sample_sizes_df %>%
  mutate(across(where(is.double), ~ round(.x, digits = 2))) %>% 
  knitr::kable()
```

| feature | value | n | rel |
| --- | --- | --- | --- |
| omicron | 0 | 205 | 0.14 |
| omicron | 1 | 1267 | 0.86 |
| sex | M | 797 | 0.54 |
| sex | W | 675 | 0.46 |
| symptoms | 0 | 798 | 0.54 |
| symptoms | 1 | 582 | 0.40 |
| symptoms | 2 | 92 | 0.06 |
| vaccination status | 1 | 494 | 0.34 |
| vaccination status | 2 | 978 | 0.66 |

## Logistic regression on viral load (Model 2)

```
# Perform final logistic regression with shrinked data set
glm_load <- glm(`test result` ~  `viral load` + sex, data = df_logit, family = binomial(link='logit'))

df_coefs_load <- as.data.frame(summary(glm_load)$coefficients) %>% as_tibble(rownames = "Feature")

odds_ratio_load <- data.frame(odds_ratio = exp(coefficients(glm_load)[-1])) %>% as_tibble(rownames = "Feature")
odds_ratio_ci_load <- data.frame(exp(confint.default(glm_load)[-1,]), check.names = F) %>% as_tibble(rownames = "Feature")

# Store odds ratios and p values of lasso regression

odds_ratio_load %>% inner_join(odds_ratio_ci_load) %>% inner_join(df_coefs_load) %>%
  writexl::write_xlsx(path = file.path(res_dir, "lasso_coefs_odds_ratios_load.xlsx"))

odds_ratio_ci_load <- odds_ratio_ci_load %>% as.data.frame()

# Plot odds ratios and CI

pdf(file.path(plt_dir, "odds_ratio_load.pdf"), width = 20, height = 7)
par(mgp = c(2.5,1,0), font.lab = 2, mfrow=c(1,1), mar = c(4.1, 4.1, 0.2, 0.2))
plot(seq_len(nrow(odds_ratio_load)), odds_ratio_load %>% pull(odds_ratio),
     ylim = c(-1,6), pch = 20, cex = 3, xaxt = "n", xlab = "coefficients",  ylab = "odds ratio")
sapply(seq_len(nrow(odds_ratio_load)), function(i){
  arrows(x0=i, y0=odds_ratio_ci_load[i,2], x1=i, y1=odds_ratio_ci_load[i,3], code=3, col="black", lwd=2, angle=90, length=0.25)
})
axis(side = 1, at = seq_len(nrow(odds_ratio_load)), labels = odds_ratio_load %>% pull(Feature))
abline(h = 1, lty = 2)
dev.off()

par(mgp = c(2.5,1,0), font.lab = 2, mfrow=c(1,1), mar = c(4.1, 4.1, 0.2, 0.2))
plot(seq_len(nrow(odds_ratio_load)), odds_ratio_load %>% pull(odds_ratio),
     ylim = c(-1,6), pch = 20, cex = 3, xaxt = "n", xlab = "coefficients",  ylab = "odds ratio")
sapply(seq_len(nrow(odds_ratio_load)), function(i){
  arrows(x0=i, y0=odds_ratio_ci_load[i,2], x1=i, y1=odds_ratio_ci_load[i,3], code=3, col="black", lwd=2, angle=90, length=0.25)
})
axis(side = 1, at = seq_len(nrow(odds_ratio_load)), labels = odds_ratio_load %>% pull(Feature))
abline(h = 1, lty = 2)
```

**Odds ratio table**

```
padj_df <- data.frame(Feature = df_coefs_load$Feature, 
                      p.adj = p.adjust(df_coefs_load$`Pr(>|z|)`, method = "BY"))

sum_odds_ratio_table_load <- odds_ratio_load %>% 
                        inner_join(odds_ratio_ci_load) %>% 
                        inner_join(df_coefs_load) %>%
                        inner_join(padj_df)

sum_odds_ratio_table_load %>% 
  mutate(across(where(is.double), 
                ~ format(.x, digits = 3, scientific = TRUE))) %>% 
  knitr::kable()
```

| Feature | odds\_ratio | 2.5 % | 97.5 % | Estimate | Std. Error | z value | Pr(>|z|) | p.adj |
| --- | --- | --- | --- | --- | --- | --- | --- | --- |
| `viral load` | 2.71e+00 | 2.45e+00 | 3.01e+00 | 9.98e-01 | 5.25e-02 | 1.9e+01 | 2.04e-80 | 5.60e-80 |
| sexW | 1.20e+00 | 9.10e-01 | 1.59e+00 | 1.84e-01 | 1.42e-01 | 1.3e+00 | 1.95e-01 | 3.58e-01 |

## Logistic regression on symptoms (Model 3)

```
# Perform final logistic regression with shrinked data set
glm_symp <- glm(`test result` ~  symptoms + sex, data = df_logit, family = binomial(link='logit'))

df_coefs_symp <- as.data.frame(summary(glm_symp)$coefficients) %>% as_tibble(rownames = "Feature")

odds_ratio_symp <- data.frame(odds_ratio = exp(coefficients(glm_symp)[-1])) %>% as_tibble(rownames = "Feature")
odds_ratio_ci_symp <- data.frame(exp(confint.default(glm_symp)[-1,]), check.names = F) %>% as_tibble(rownames = "Feature")

# Store odds ratios and p values of lasso regression

odds_ratio_symp %>% inner_join(odds_ratio_ci_symp) %>% inner_join(df_coefs_symp) %>%
  writexl::write_xlsx(path = file.path(res_dir, "lasso_coefs_odds_ratios_symp.xlsx"))

odds_ratio_ci_symp <- odds_ratio_ci_symp %>% as.data.frame()

# Plot odds ratios and CI

pdf(file.path(plt_dir, "odds_ratio_symp.pdf"), width = 20, height = 7)
par(mgp = c(2.5,1,0), font.lab = 2, mfrow=c(1,1), mar = c(4.1, 4.1, 0.2, 0.2))
plot(seq_len(nrow(odds_ratio_symp)), odds_ratio_symp %>% pull(odds_ratio),
     ylim = c(-1,6), pch = 20, cex = 3, xaxt = "n", xlab = "coefficients",  ylab = "odds ratio")
sapply(seq_len(nrow(odds_ratio_symp)), function(i){
  arrows(x0=i, y0=odds_ratio_ci_symp[i,2], x1=i, y1=odds_ratio_ci_symp[i,3], code=3, col="black", lwd=2, angle=90, length=0.25)
})
axis(side = 1, at = seq_len(nrow(odds_ratio_symp)), labels = odds_ratio_symp %>% pull(Feature))
abline(h = 1, lty = 2)
dev.off()

par(mgp = c(2.5,1,0), font.lab = 2, mfrow=c(1,1), mar = c(4.1, 4.1, 0.2, 0.2))
plot(seq_len(nrow(odds_ratio_symp)), odds_ratio_symp %>% pull(odds_ratio),
     ylim = c(-1,6), pch = 20, cex = 3, xaxt = "n", xlab = "coefficients",  ylab = "odds ratio")
sapply(seq_len(nrow(odds_ratio_symp)), function(i){
  arrows(x0=i, y0=odds_ratio_ci_symp[i,2], x1=i, y1=odds_ratio_ci_symp[i,3], code=3, col="black", lwd=2, angle=90, length=0.25)
})
axis(side = 1, at = seq_len(nrow(odds_ratio_symp)), labels = odds_ratio_symp %>% pull(Feature))
abline(h = 1, lty = 2)
```

**Odds ratio table**

```
padj_df <- data.frame(Feature = df_coefs_symp$Feature, 
                      p.adj = p.adjust(df_coefs_symp$`Pr(>|z|)`, method = "BY"))

sum_odds_ratio_table_symp <- odds_ratio_symp %>% 
                        inner_join(odds_ratio_ci_symp) %>% 
                        inner_join(df_coefs_symp) %>%
                        inner_join(padj_df)

sum_odds_ratio_table_symp %>% 
  mutate(across(where(is.double), 
                ~ format(.x, digits = 3, scientific = TRUE))) %>% 
  knitr::kable()
```

| Feature | odds\_ratio | 2.5 % | 97.5 % | Estimate | Std. Error | z value | Pr(>|z|) | p.adj |
| --- | --- | --- | --- | --- | --- | --- | --- | --- |
| symptoms1 | 4.35e+00 | 3.43e+00 | 5.51e+00 | 1.47e+00 | 1.21e-01 | 1.21e+01 | 6.44e-34 | 2.68e-33 |
| symptoms2 | 3.15e+00 | 2.02e+00 | 4.92e+00 | 1.15e+00 | 2.28e-01 | 5.05e+00 | 4.52e-07 | 1.26e-06 |
| sexW | 1.10e+00 | 8.73e-01 | 1.38e+00 | 9.18e-02 | 1.16e-01 | 7.89e-01 | 4.30e-01 | 8.96e-01 |

## Logistic regression on omicron infection (Model 4)

```
# Perform final logistic regression with shrinked data set
glm_omic <- glm(`test result` ~  omicron, data = df_logit, family = binomial(link='logit'))

df_coefs_omic <- as.data.frame(summary(glm_omic)$coefficients) %>% as_tibble(rownames = "Feature")

odds_ratio_omic <- data.frame(odds_ratio = exp(coefficients(glm_omic)[-1])) %>% as_tibble(rownames = "Feature")
odds_ratio_ci_omic <- data.frame(exp(confint.default(glm_omic)), check.names = F)[-1,] %>% as_tibble(rownames = "Feature")

# Store odds ratios and p values of lasso regression

odds_ratio_omic %>% inner_join(odds_ratio_ci_omic) %>% inner_join(df_coefs_omic) %>%
  writexl::write_xlsx(path = file.path(res_dir, "lasso_coefs_odds_ratios_omic.xlsx"))

odds_ratio_ci_omic <- odds_ratio_ci_omic %>% as.data.frame()

# Plot odds ratios and CI

pdf(file.path(plt_dir, "odds_ratio_omic.pdf"), width = 20, height = 7)
par(mgp = c(2.5,1,0), font.lab = 2, mfrow=c(1,1), mar = c(4.1, 4.1, 0.2, 0.2))
plot(seq_len(nrow(odds_ratio_omic)), odds_ratio_omic %>% pull(odds_ratio),
     ylim = c(-1,6), pch = 20, cex = 3, xaxt = "n", xlab = "coefficients",  ylab = "odds ratio")
sapply(seq_len(nrow(odds_ratio_omic)), function(i){
  arrows(x0=i, y0=odds_ratio_ci_omic[i,2], x1=i, y1=odds_ratio_ci_omic[i,3], code=3, col="black", lwd=2, angle=90, length=0.25)
})
axis(side = 1, at = seq_len(nrow(odds_ratio_omic)), labels = odds_ratio_omic %>% pull(Feature))
abline(h = 1, lty = 2)
dev.off()

par(mgp = c(2.5,1,0), font.lab = 2, mfrow=c(1,1), mar = c(4.1, 4.1, 0.2, 0.2))
plot(seq_len(nrow(odds_ratio_omic)), odds_ratio_omic %>% pull(odds_ratio),
     ylim = c(-1,6), pch = 20, cex = 3, xaxt = "n", xlab = "coefficients",  ylab = "odds ratio")
sapply(seq_len(nrow(odds_ratio_omic)), function(i){
  arrows(x0=i, y0=odds_ratio_ci_omic[i,2], x1=i, y1=odds_ratio_ci_omic[i,3], code=3, col="black", lwd=2, angle=90, length=0.25)
})
axis(side = 1, at = seq_len(nrow(odds_ratio_omic)), labels = odds_ratio_omic %>% pull(Feature))
abline(h = 1, lty = 2)
```

**Odds ratio table**

```
padj_df <- data.frame(Feature = df_coefs_omic$Feature, 
                      p.adj = p.adjust(df_coefs_omic$`Pr(>|z|)`, method = "BY"))

sum_odds_ratio_table_omic <- odds_ratio_omic %>% 
                        inner_join(odds_ratio_ci_omic) %>% 
                        inner_join(df_coefs_omic) %>%
                        inner_join(padj_df)

sum_odds_ratio_table_omic %>% 
  mutate(across(where(is.double), 
                ~ format(.x, digits = 3, scientific = TRUE))) %>% 
  knitr::kable()
```

| Feature | odds\_ratio | 2.5 % | 97.5 % | Estimate | Std. Error | z value | Pr(>|z|) | p.adj |
| --- | --- | --- | --- | --- | --- | --- | --- | --- |
| omicron | 8.19e-01 | 6.04e-01 | 1.11e+00 | -1.99e-01 | 1.55e-01 | -1.28e+00 | 2e-01 | 2.99e-01 |

## Logistic regression on vaccination status (Model 5)

```
# Perform final logistic regression with shrinked data set
glm_vaccs <- glm(`test result` ~  `vaccination status`, data = df_logit, family = binomial(link='logit'))

df_coefs_vaccs <- as.data.frame(summary(glm_vaccs)$coefficients) %>% as_tibble(rownames = "Feature")

odds_ratio_vaccs <- data.frame(odds_ratio = exp(coefficients(glm_vaccs)[-1])) %>% as_tibble(rownames = "Feature")
odds_ratio_ci_vaccs <- data.frame(exp(confint.default(glm_vaccs)), check.names = F)[-1,] %>% as_tibble(rownames = "Feature")

# Store odds ratios and p values of lasso regression

odds_ratio_vaccs %>% inner_join(odds_ratio_ci_vaccs) %>% inner_join(df_coefs_vaccs) %>%
  writexl::write_xlsx(path = file.path(res_dir, "lasso_coefs_odds_ratios_vaccs.xlsx"))

odds_ratio_ci_vaccs <- odds_ratio_ci_vaccs %>% as.data.frame()

# Plot odds ratios and CI

pdf(file.path(plt_dir, "odds_ratio_vaccs.pdf"), width = 20, height = 7)
par(mgp = c(2.5,1,0), font.lab = 2, mfrow=c(1,1), mar = c(4.1, 4.1, 0.2, 0.2))
plot(seq_len(nrow(odds_ratio_vaccs)), odds_ratio_vaccs %>% pull(odds_ratio),
     ylim = c(-1,6), pch = 20, cex = 3, xaxt = "n", xlab = "coefficients",  ylab = "odds ratio")
sapply(seq_len(nrow(odds_ratio_vaccs)), function(i){
  arrows(x0=i, y0=odds_ratio_ci_vaccs[i,2], x1=i, y1=odds_ratio_ci_vaccs[i,3], code=3, col="black", lwd=2, angle=90, length=0.25)
})
axis(side = 1, at = seq_len(nrow(odds_ratio_vaccs)), labels = odds_ratio_vaccs %>% pull(Feature))
abline(h = 1, lty = 2)
dev.off()

par(mgp = c(2.5,1,0), font.lab = 2, mfrow=c(1,1), mar = c(4.1, 4.1, 0.2, 0.2))
plot(seq_len(nrow(odds_ratio_vaccs)), odds_ratio_vaccs %>% pull(odds_ratio),
     ylim = c(-1,6), pch = 20, cex = 3, xaxt = "n", xlab = "coefficients",  ylab = "odds ratio")
sapply(seq_len(nrow(odds_ratio_vaccs)), function(i){
  arrows(x0=i, y0=odds_ratio_ci_vaccs[i,2], x1=i, y1=odds_ratio_ci_vaccs[i,3], code=3, col="black", lwd=2, angle=90, length=0.25)
})
axis(side = 1, at = seq_len(nrow(odds_ratio_vaccs)), labels = odds_ratio_vaccs %>% pull(Feature))
abline(h = 1, lty = 2)
```

**Odds ratio table**

```
padj_df <- data.frame(Feature = df_coefs_vaccs$Feature, 
                      p.adj = p.adjust(df_coefs_vaccs$`Pr(>|z|)`, method = "BY"))

sum_odds_ratio_table_vaccs <- odds_ratio_vaccs %>% 
                        inner_join(odds_ratio_ci_vaccs) %>% 
                        inner_join(df_coefs_vaccs) %>%
                        inner_join(padj_df)

sum_odds_ratio_table_vaccs %>% 
  mutate(across(where(is.double), 
                ~ format(.x, digits = 3, scientific = TRUE))) %>% 
  knitr::kable()
```

| Feature | odds\_ratio | 2.5 % | 97.5 % | Estimate | Std. Error | z value | Pr(>|z|) | p.adj |
| --- | --- | --- | --- | --- | --- | --- | --- | --- |
| `vaccination status`2 | 5.96e-01 | 4.76e-01 | 7.45e-01 | -5.18e-01 | 1.14e-01 | -4.53e+00 | 5.94e-06 | 1.78e-05 |

## Logistic regression on symptoms + viral load (Model 6)

```
glm_sym_load <- glm(`test result` ~  symptoms + `viral load` + sex, data = df_logit, family = binomial(link='logit'))

df_coefs_sym_load <- as.data.frame(summary(glm_sym_load)$coefficients) %>% as_tibble(rownames = "Feature")

odds_ratio_sym_load <- data.frame(odds_ratio = exp(coefficients(glm_sym_load)[-1])) %>% as_tibble(rownames = "Feature")
odds_ratio_ci_sym_load <- data.frame(exp(confint.default(glm_sym_load)[-1,]), check.names = F) %>% as_tibble(rownames = "Feature")

odds_ratio_sym_load %>% inner_join(odds_ratio_ci_sym_load) %>% inner_join(df_coefs_sym_load) %>%
  writexl::write_xlsx(path = file.path(res_dir, "lasso_coefs_odds_ratios_sym_load.xlsx"))

odds_ratio_ci_sym_load <- odds_ratio_ci_sym_load %>% as.data.frame()

# Plot odds ratios and CI

pdf(file.path(plt_dir, "odds_ratio_sym_load.pdf"), width = 20, height = 7)
par(mgp = c(2.5,1,0), font.lab = 2, mfrow=c(1,1), mar = c(4.1, 4.1, 0.2, 0.2))
plot(seq_len(nrow(odds_ratio_sym_load)), odds_ratio_sym_load %>% pull(odds_ratio),
     ylim = c(-1,6), pch = 20, cex = 3, xaxt = "n", xlab = "coefficients",  ylab = "odds ratio")
sapply(seq_len(nrow(odds_ratio_sym_load)), function(i){
  arrows(x0=i, y0=odds_ratio_ci_sym_load[i,2], x1=i, y1=odds_ratio_ci_sym_load[i,3], code=3, col="black", lwd=2, angle=90, length=0.25)
})
axis(side = 1, at = seq_len(nrow(odds_ratio_sym_load)), labels = odds_ratio_sym_load %>% pull(Feature))
abline(h = 1, lty = 2)
dev.off()

par(mgp = c(2.5,1,0), font.lab = 2, mfrow=c(1,1), mar = c(4.1, 4.1, 0.2, 0.2))
plot(seq_len(nrow(odds_ratio_sym_load)), odds_ratio_sym_load %>% pull(odds_ratio),
     ylim = c(-1,6), pch = 20, cex = 3, xaxt = "n", xlab = "coefficients",  ylab = "odds ratio")
sapply(seq_len(nrow(odds_ratio_sym_load)), function(i){
  arrows(x0=i, y0=odds_ratio_ci_sym_load[i,2], x1=i, y1=odds_ratio_ci_sym_load[i,3], code=3, col="black", lwd=2, angle=90, length=0.25)
})
axis(side = 1, at = seq_len(nrow(odds_ratio_sym_load)), labels = odds_ratio_sym_load %>% pull(Feature))
abline(h = 1, lty = 2)
```

**Odds ratio table**

```
padj_df <- data.frame(Feature = df_coefs_sym_load$Feature, 
                      p.adj = p.adjust(df_coefs_sym_load$`Pr(>|z|)`, method = "BY"))

sum_odds_ratio_table_sym_load <- odds_ratio_sym_load %>% 
                        inner_join(odds_ratio_ci_sym_load) %>% 
                        inner_join(df_coefs_sym_load) %>%
                        inner_join(padj_df)

sum_odds_ratio_table_sym_load %>% 
  mutate(across(where(is.double), 
                ~ format(.x, digits = 3, scientific = TRUE))) %>% 
  knitr::kable()
```

| Feature | odds\_ratio | 2.5 % | 97.5 % | Estimate | Std. Error | z value | Pr(>|z|) | p.adj |
| --- | --- | --- | --- | --- | --- | --- | --- | --- |
| symptoms1 | 2.70e+00 | 2.01e+00 | 3.63e+00 | 9.95e-01 | 1.50e-01 | 6.62e+00 | 3.62e-11 | 1.38e-10 |
| symptoms2 | 1.51e+00 | 8.50e-01 | 2.70e+00 | 4.15e-01 | 2.95e-01 | 1.41e+00 | 1.59e-01 | 4.54e-01 |
| `viral load` | 2.59e+00 | 2.33e+00 | 2.87e+00 | 9.51e-01 | 5.34e-02 | 1.78e+01 | 6.00e-71 | 3.43e-70 |
| sexW | 1.15e+00 | 8.66e-01 | 1.53e+00 | 1.41e-01 | 1.45e-01 | 9.72e-01 | 3.31e-01 | 7.56e-01 |

## Pairwise comparison: viral load and symptoms

```
df_logit %>% 
  ggplot2::ggplot(ggplot2::aes(x = symptoms, 
                               y = `viral load`, 
                               fill = factor(`test result`))) +
  ggplot2::stat_boxplot(geom = "errorbar", lwd = 1,show.legend = FALSE, position = ggplot2::position_dodge2(0.9)) +
  ggplot2::geom_boxplot(color = "black", lwd = 1, show.legend = TRUE, outlier.size = 0, position = ggplot2::position_dodge2(0.8)) +
  ggbeeswarm::geom_quasirandom(alpha = 0.8, pch = 20, size = 1, dodge.width = .8, color="black",alpha=.5,show.legend = F) +
  ggplot2::theme_bw() +
  ggsci::scale_fill_bmj() +
  ggplot2::theme(axis.text = ggplot2::element_text(size = 14, colour = "black"),
                 axis.title = ggplot2::element_text(face = "bold", size = 12),
                 legend.title = ggplot2::element_blank(),
                 legend.text = ggplot2::element_text(size = 12),
                 legend.position = "bottom") +
  ggplot2::xlab("Typical symptoms") +
  ggplot2::ylab("viral load") +
  # ggsignif::geom_signif(comparisons = list(c("yes", "no")),
  #                       annotation = format(wt_res$p.value, scientific = T, digits = 2),
  #                       tip_length = c(0.2, 0.04)) + 
  ggplot2::scale_y_continuous(expand = expansion(mult = c(0.05, .1)))
```

```
df_test_symp <- df_logit %>% group_by(`test result`, symptoms) %>% dplyr::count() %>% 
  tidyr::pivot_wider(names_from = symptoms, values_from = n) %>% as.data.frame()

rownames(df_test_symp) <- as.character(df_test_symp[,1])
df_test_symp <- df_test_symp[,-1]

for(i in seq_len(ncol(df_test_symp))){
  fisher.test(df_test_symp[,-i][,2:1])
}
```

## Logistic regression on viral load + vaccination status (Model 7)

```
# Perform final logistic regression with shrinked data set
glm_vaccs_load <- glm(`test result` ~  `viral load` + `vaccination status` + sex, data = df_logit, family = binomial(link='logit'))

df_coefs_vaccs_load <- as.data.frame(summary(glm_vaccs_load)$coefficients) %>% as_tibble(rownames = "Feature")

odds_ratio_vaccs_load <- data.frame(odds_ratio = exp(coefficients(glm_vaccs_load)[-1])) %>% as_tibble(rownames = "Feature")
odds_ratio_ci_vaccs_load <- data.frame(exp(confint.default(glm_vaccs_load)), check.names = F)[-1,] %>% as_tibble(rownames = "Feature")

# Store odds ratios and p values of lasso regression

odds_ratio_vaccs_load %>% inner_join(odds_ratio_ci_vaccs_load) %>% inner_join(df_coefs_vaccs_load) %>%
  writexl::write_xlsx(path = file.path(res_dir, "lasso_coefs_odds_ratios_vaccs_load.xlsx"))

odds_ratio_ci_vaccs_load <- odds_ratio_ci_vaccs_load %>% as.data.frame()

# Plot odds ratios and CI

pdf(file.path(plt_dir, "odds_ratio_vaccs_load.pdf"), width = 20, height = 7)
par(mgp = c(2.5,1,0), font.lab = 2, mfrow=c(1,1), mar = c(4.1, 4.1, 0.2, 0.2))
plot(seq_len(nrow(odds_ratio_vaccs_load)), odds_ratio_vaccs_load %>% pull(odds_ratio),
     ylim = c(-1,6), pch = 20, cex = 3, xaxt = "n", xlab = "coefficients",  ylab = "odds ratio")
sapply(seq_len(nrow(odds_ratio_vaccs_load)), function(i){
  arrows(x0=i, y0=odds_ratio_ci_vaccs_load[i,2], x1=i, y1=odds_ratio_ci_vaccs_load[i,3], code=3, col="black", lwd=2, angle=90, length=0.25)
})
axis(side = 1, at = seq_len(nrow(odds_ratio_vaccs_load)), labels = odds_ratio_vaccs_load %>% pull(Feature))
abline(h = 1, lty = 2)
dev.off()

par(mgp = c(2.5,1,0), font.lab = 2, mfrow=c(1,1), mar = c(4.1, 4.1, 0.2, 0.2))
plot(seq_len(nrow(odds_ratio_vaccs_load)), odds_ratio_vaccs_load %>% pull(odds_ratio),
     ylim = c(-1,6), pch = 20, cex = 3, xaxt = "n", xlab = "coefficients",  ylab = "odds ratio")
sapply(seq_len(nrow(odds_ratio_vaccs_load)), function(i){
  arrows(x0=i, y0=odds_ratio_ci_vaccs_load[i,2], x1=i, y1=odds_ratio_ci_vaccs_load[i,3], code=3, col="black", lwd=2, angle=90, length=0.25)
})
axis(side = 1, at = seq_len(nrow(odds_ratio_vaccs_load)), labels = odds_ratio_vaccs_load %>% pull(Feature))
abline(h = 1, lty = 2)
```

**Odds ratio table**

```
padj_df <- data.frame(Feature = df_coefs_vaccs_load$Feature, 
                      p.adj = p.adjust(df_coefs_vaccs_load$`Pr(>|z|)`, method = "BY"))

sum_odds_ratio_table_vaccs_load <- odds_ratio_vaccs_load %>% 
                        inner_join(odds_ratio_ci_vaccs_load) %>% 
                        inner_join(df_coefs_vaccs_load) %>%
                        inner_join(padj_df)

sum_odds_ratio_table_vaccs_load %>% 
  mutate(across(where(is.double), 
                ~ format(.x, digits = 3, scientific = TRUE))) %>% 
  knitr::kable()
```

| Feature | odds\_ratio | 2.5 % | 97.5 % | Estimate | Std. Error | z value | Pr(>|z|) | p.adj |
| --- | --- | --- | --- | --- | --- | --- | --- | --- |
| `viral load` | 2.73e+00 | 2.46e+00 | 3.03e+00 | 1.00e+00 | 5.31e-02 | 1.89e+01 | 1.20e-79 | 1.00e-78 |
| `vaccination status`2 | 5.51e-01 | 4.11e-01 | 7.38e-01 | -5.96e-01 | 1.49e-01 | -3.99e+00 | 6.57e-05 | 1.82e-04 |
| sexW | 1.19e+00 | 8.96e-01 | 1.57e+00 | 1.71e-01 | 1.43e-01 | 1.19e+00 | 2.32e-01 | 4.84e-01 |

## Logistic regression on vaccination status + symptoms (Model 8)

```
# Perform final logistic regression with shrinked data set
glm_vaccs_sym <- glm(`test result` ~  `vaccination status` + symptoms + sex, data = df_logit, family = binomial(link='logit'))

df_coefs_vaccs_sym <- as.data.frame(summary(glm_vaccs_sym)$coefficients) %>% as_tibble(rownames = "Feature")

odds_ratio_vaccs_sym <- data.frame(odds_ratio = exp(coefficients(glm_vaccs_sym)[-1])) %>% as_tibble(rownames = "Feature")
odds_ratio_ci_vaccs_sym <- data.frame(exp(confint.default(glm_vaccs_sym)), check.names = F)[-1,] %>% as_tibble(rownames = "Feature")

# Store odds ratios and p values of lasso regression

odds_ratio_vaccs_sym %>% inner_join(odds_ratio_ci_vaccs_sym) %>% inner_join(df_coefs_vaccs_sym) %>%
  writexl::write_xlsx(path = file.path(res_dir, "lasso_coefs_odds_ratios_vaccs_sym.xlsx"))

odds_ratio_ci_vaccs_sym <- odds_ratio_ci_vaccs_sym %>% as.data.frame()

# Plot odds ratios and CI

pdf(file.path(plt_dir, "odds_ratio_vaccs_sym.pdf"), width = 20, height = 7)
par(mgp = c(2.5,1,0), font.lab = 2, mfrow=c(1,1), mar = c(4.1, 4.1, 0.2, 0.2))
plot(seq_len(nrow(odds_ratio_vaccs_sym)), odds_ratio_vaccs_sym %>% pull(odds_ratio),
     ylim = c(-1,6), pch = 20, cex = 3, xaxt = "n", xlab = "coefficients",  ylab = "odds ratio")
sapply(seq_len(nrow(odds_ratio_vaccs_sym)), function(i){
  arrows(x0=i, y0=odds_ratio_ci_vaccs_sym[i,2], x1=i, y1=odds_ratio_ci_vaccs_sym[i,3], code=3, col="black", lwd=2, angle=90, length=0.25)
})
axis(side = 1, at = seq_len(nrow(odds_ratio_vaccs_sym)), labels = odds_ratio_vaccs_sym %>% pull(Feature))
abline(h = 1, lty = 2)
dev.off()

par(mgp = c(2.5,1,0), font.lab = 2, mfrow=c(1,1), mar = c(4.1, 4.1, 0.2, 0.2))
plot(seq_len(nrow(odds_ratio_vaccs_sym)), odds_ratio_vaccs_sym %>% pull(odds_ratio),
     ylim = c(-1,6), pch = 20, cex = 3, xaxt = "n", xlab = "coefficients",  ylab = "odds ratio")
sapply(seq_len(nrow(odds_ratio_vaccs_sym)), function(i){
  arrows(x0=i, y0=odds_ratio_ci_vaccs_sym[i,2], x1=i, y1=odds_ratio_ci_vaccs_sym[i,3], code=3, col="black", lwd=2, angle=90, length=0.25)
})
axis(side = 1, at = seq_len(nrow(odds_ratio_vaccs_sym)), labels = odds_ratio_vaccs_sym %>% pull(Feature))
abline(h = 1, lty = 2)
```

**Odds ratio table**

```
padj_df <- data.frame(Feature = df_coefs_vaccs_sym$Feature, 
                      p.adj = p.adjust(df_coefs_vaccs_sym$`Pr(>|z|)`, method = "BY"))

sum_odds_ratio_table_vaccs_sym <- odds_ratio_vaccs_sym %>% 
                        inner_join(odds_ratio_ci_vaccs_sym) %>% 
                        inner_join(df_coefs_vaccs_sym) %>%
                        inner_join(padj_df)

sum_odds_ratio_table_vaccs_sym %>% 
  mutate(across(where(is.double), 
                ~ format(.x, digits = 3, scientific = TRUE))) %>% 
  knitr::kable()
```

| Feature | odds\_ratio | 2.5 % | 97.5 % | Estimate | Std. Error | z value | Pr(>|z|) | p.adj |
| --- | --- | --- | --- | --- | --- | --- | --- | --- |
| `vaccination status`2 | 9.43e-01 | 7.36e-01 | 1.21e+00 | -5.89e-02 | 1.27e-01 | -4.65e-01 | 6.42e-01 | 1.00e+00 |
| symptoms1 | 4.27e+00 | 3.32e+00 | 5.47e+00 | 1.45e+00 | 1.27e-01 | 1.14e+01 | 4.69e-30 | 5.35e-29 |
| symptoms2 | 3.11e+00 | 1.99e+00 | 4.88e+00 | 1.14e+00 | 2.29e-01 | 4.96e+00 | 7.23e-07 | 2.75e-06 |
| sexW | 1.10e+00 | 8.73e-01 | 1.38e+00 | 9.18e-02 | 1.16e-01 | 7.89e-01 | 4.30e-01 | 1.00e+00 |

# Summarized models

```
sum_odds_ratio_table_load <- sum_odds_ratio_table_load %>% 
                                mutate(type = if_else(Feature %in% c("`viral load`", "symptoms1", "symptoms2", "`vaccination status`2"), 
                                                      true = "covariate", false = "confounding"),
                                       type = factor(type, levels = c("covariate", "confounding"))) %>% 
                                arrange(type, Feature)

sum_odds_ratio_table_omic <- sum_odds_ratio_table_omic %>% 
                                mutate(type = if_else(Feature %in% c("`viral load`", "symptoms1", "symptoms2", "omicron", "`vaccination status`2"), 
                                                      true = "covariate", false = "confounding"),
                                       type = factor(type, levels = c("covariate", "confounding"))) %>% 
                                arrange(type, Feature)  %>%
                                mutate(Feature = case_when(Feature == "symptoms1" ~ "typical symptomatic", 
                                                           Feature == "symptoms2" ~ "atypical symptomatic",
                                                           TRUE ~ Feature))

sum_odds_ratio_table_symp <- sum_odds_ratio_table_symp %>% 
                                mutate(type = if_else(Feature %in% c("`viral load`", "symptoms1", "symptoms2", "`vaccination status`2"), 
                                                      true = "covariate", false = "confounding"),
                                       type = factor(type, levels = c("covariate", "confounding"))) %>% 
                                arrange(type, Feature) %>%
                                mutate(Feature = case_when(Feature == "symptoms1" ~ "typical symptomatic", 
                                                           Feature == "symptoms2" ~ "atypical symptomatic",
                                                           TRUE ~ Feature))
                                                           
sum_odds_ratio_table_vaccs <- sum_odds_ratio_table_vaccs %>% 
                                mutate(type = if_else(Feature %in% c("`viral load`", "symptoms1", "symptoms2", "omicron", "`vaccination status`2"), 
                                                      true = "covariate", false = "confounding"),
                                       type = factor(type, levels = c("covariate", "confounding"))) %>% 
                                arrange(type, Feature) %>%
                                mutate(Feature = case_when(Feature == "symptoms1" ~ "typical symptomatic", 
                                                           Feature == "symptoms2" ~ "atypical symptomatic",
                                                           TRUE ~ Feature))
                                                           
sum_odds_ratio_table_sym_load <- sum_odds_ratio_table_sym_load %>% 
                                mutate(type = if_else(Feature %in% c("`viral load`", "symptoms1", "symptoms2", "`vaccination status`2"), 
                                                      true = "covariate", false = "confounding"),
                                       type = factor(type, levels = c("covariate", "confounding"))) %>% 
                                arrange(type, Feature) %>%
                                mutate(Feature = case_when(Feature == "symptoms1" ~ "typical symptomatic", 
                                                           Feature == "symptoms2" ~ "atypical symptomatic",
                                                           TRUE ~ Feature))

sum_odds_ratio_table_vaccs_load <- sum_odds_ratio_table_vaccs_load %>% 
                                mutate(type = if_else(Feature %in% c("`viral load`", "symptoms1", "symptoms2", "omicron", "`vaccination status`2"), 
                                                      true = "covariate", false = "confounding"),
                                       type = factor(type, levels = c("covariate", "confounding"))) %>% 
                                arrange(type, Feature)                                
                                                           
sum_odds_ratio_table_vaccs_sym <- sum_odds_ratio_table_vaccs_sym %>% 
                                mutate(type = if_else(Feature %in% c("`viral load`", "symptoms1", "symptoms2", "omicron", "`vaccination status`2"), 
                                                      true = "covariate", false = "confounding"),
                                       type = factor(type, levels = c("covariate", "confounding"))) %>% 
                                arrange(type, Feature) %>%
                                mutate(Feature = case_when(Feature == "symptoms1" ~ "typical symptomatic", 
                                                           Feature == "symptoms2" ~ "atypical symptomatic",
                                                           TRUE ~ Feature))                                                         
                                                           

sum_odds_ratio_table <- sum_odds_ratio_table %>% 
                                mutate(type = if_else(Feature %in% c("`viral load`", "symptoms1", "symptoms2", "omicron", "`vaccination status`2"), 
                                                      true = "covariate", false = "confounding"),
                                       type = factor(type, levels = c("covariate", "confounding"))) %>% 
                                arrange(type, Feature) %>%
                                mutate(Feature = case_when(Feature == "symptoms1" ~ "typical symptomatic", 
                                                           Feature == "symptoms2" ~ "atypical symptomatic",
                                                           TRUE ~ Feature))   

list_sum_tables <- list(sum_odds_ratio_table,
                        sum_odds_ratio_table_load, 
                        sum_odds_ratio_table_symp,
                        sum_odds_ratio_table_omic,
                        sum_odds_ratio_table_vaccs,
                        sum_odds_ratio_table_sym_load,
                        sum_odds_ratio_table_vaccs_load,
                        sum_odds_ratio_table_vaccs_sym)
                                                
sum_table_test_results <- do.call("rbind", list_sum_tables)

sum_table_test_results <- sum_table_test_results %>% dplyr::rename("p value" = `Pr(>|z|)`)
sum_table_test_results$p.adj <- p.adjust(sum_table_test_results$`p value`, method = "BY")

index_line <- cumsum(unlist(lapply(list_sum_tables, nrow))) + 1
                       
knitr::kable(sum_table_test_results %>% 
              mutate(across(where(is.double), 
                    ~ format(.x, digits = 3, scientific = TRUE)))) %>%
  kable_paper("striped", full_width = F) %>%
   kableExtra::pack_rows(group_label = "Model 1", 1, index_line[1],hline_after = T, indent = F) %>% 
   kableExtra::pack_rows(group_label = "Model 2", start_row = index_line[1], end_row = index_line[2], hline_after = T, indent = F) %>% 
   kableExtra::pack_rows(group_label = "Model 3", start_row = index_line[2], end_row = index_line[3]-1, hline_after = T, indent = F) %>%
   kableExtra::pack_rows(group_label = "Model 4", start_row = index_line[3], end_row = index_line[4]-1, hline_after = T, indent = F) %>%
   kableExtra::pack_rows(group_label = "Model 5", start_row = index_line[4], end_row = index_line[5]-1, hline_after = T, indent = F) %>%
   kableExtra::pack_rows(group_label = "Model 6", start_row = index_line[5], end_row = index_line[6]-1, hline_after = T, indent = F) %>%
   kableExtra::pack_rows(group_label = "Model 7", start_row = index_line[6], end_row = index_line[7]-1, hline_after = T, indent = F) %>%
   kableExtra::pack_rows(group_label = "Model 8", start_row = index_line[7], end_row = index_line[8]-1, hline_after = T, indent = F) %>%
   kable_styling()%>%
   row_spec(c(1:5, index_line[1], index_line[2] + 0:1, index_line[3], index_line[4],
              index_line[5] + 0:2, index_line[6] + 0:1, index_line[7] + 0:2), 
              bold=T, hline_after = T)
```

| Feature | odds\_ratio | 2.5 % | 97.5 % | Estimate | Std. Error | z value | p value | p.adj | type |
| --- | --- | --- | --- | --- | --- | --- | --- | --- | --- |
| **Model 1** | | | | | | | | | |
| `vaccination status`2 | 7.58e-01 | 5.47e-01 | 1.05e+00 | -2.77e-01 | 1.66e-01 | -1.67e+00 | 9.57e-02 | 6.67e-01 | covariate |
| `viral load` | 2.62e+00 | 2.35e+00 | 2.91e+00 | 9.61e-01 | 5.40e-02 | 1.78e+01 | 8.61e-71 | 1.95e-69 | covariate |
| omicron | 8.62e-01 | 5.71e-01 | 1.30e+00 | -1.48e-01 | 2.10e-01 | -7.04e-01 | 4.81e-01 | 1.00e+00 | covariate |
| typical symptomatic | 2.42e+00 | 1.77e+00 | 3.31e+00 | 8.86e-01 | 1.59e-01 | 5.55e+00 | 2.80e-08 | 3.18e-07 | covariate |
| atypical symptomatic | 1.39e+00 | 7.73e-01 | 2.48e+00 | 3.26e-01 | 2.98e-01 | 1.09e+00 | 2.74e-01 | 1.00e+00 | covariate |
| sexW | 1.15e+00 | 8.65e-01 | 1.53e+00 | 1.40e-01 | 1.45e-01 | 9.62e-01 | 3.36e-01 | 1.00e+00 | confounding |
| **Model 2** | | | | | | | | | |
| `viral load` | 2.71e+00 | 2.45e+00 | 3.01e+00 | 9.98e-01 | 5.25e-02 | 1.90e+01 | 2.04e-80 | 1.84e-78 | covariate |
| sexW | 1.20e+00 | 9.10e-01 | 1.59e+00 | 1.84e-01 | 1.42e-01 | 1.30e+00 | 1.95e-01 | 1.00e+00 | confounding |
| **Model 3** | | | | | | | | | |
| typical symptomatic | 4.35e+00 | 3.43e+00 | 5.51e+00 | 1.47e+00 | 1.21e-01 | 1.21e+01 | 6.44e-34 | 1.17e-32 | covariate |
| atypical symptomatic | 3.15e+00 | 2.02e+00 | 4.92e+00 | 1.15e+00 | 2.28e-01 | 5.05e+00 | 4.52e-07 | 4.55e-06 | covariate |
| sexW | 1.10e+00 | 8.73e-01 | 1.38e+00 | 9.18e-02 | 1.16e-01 | 7.89e-01 | 4.30e-01 | 1.00e+00 | confounding |
| **Model 4** | | | | | | | | | |
| omicron | 8.19e-01 | 6.04e-01 | 1.11e+00 | -1.99e-01 | 1.55e-01 | -1.28e+00 | 2.00e-01 | 1.00e+00 | covariate |
| **Model 5** | | | | | | | | | |
| `vaccination status`2 | 5.96e-01 | 4.76e-01 | 7.45e-01 | -5.18e-01 | 1.14e-01 | -4.53e+00 | 5.94e-06 | 4.89e-05 | covariate |
| **Model 6** | | | | | | | | | |
| `viral load` | 2.59e+00 | 2.33e+00 | 2.87e+00 | 9.51e-01 | 5.34e-02 | 1.78e+01 | 6.00e-71 | 1.81e-69 | covariate |
| typical symptomatic | 2.70e+00 | 2.01e+00 | 3.63e+00 | 9.95e-01 | 1.50e-01 | 6.62e+00 | 3.62e-11 | 4.68e-10 | covariate |
| atypical symptomatic | 1.51e+00 | 8.50e-01 | 2.70e+00 | 4.15e-01 | 2.95e-01 | 1.41e+00 | 1.59e-01 | 1.00e+00 | covariate |
| sexW | 1.15e+00 | 8.66e-01 | 1.53e+00 | 1.41e-01 | 1.45e-01 | 9.72e-01 | 3.31e-01 | 1.00e+00 | confounding |
| **Model 7** | | | | | | | | | |
| `vaccination status`2 | 5.51e-01 | 4.11e-01 | 7.38e-01 | -5.96e-01 | 1.49e-01 | -3.99e+00 | 6.57e-05 | 4.96e-04 | covariate |
| `viral load` | 2.73e+00 | 2.46e+00 | 3.03e+00 | 1.00e+00 | 5.31e-02 | 1.89e+01 | 1.20e-79 | 5.45e-78 | covariate |
| sexW | 1.19e+00 | 8.96e-01 | 1.57e+00 | 1.71e-01 | 1.43e-01 | 1.19e+00 | 2.32e-01 | 1.00e+00 | confounding |
| **Model 8** | | | | | | | | | |
| `vaccination status`2 | 9.43e-01 | 7.36e-01 | 1.21e+00 | -5.89e-02 | 1.27e-01 | -4.65e-01 | 6.42e-01 | 1.00e+00 | covariate |
| typical symptomatic | 4.27e+00 | 3.32e+00 | 5.47e+00 | 1.45e+00 | 1.27e-01 | 1.14e+01 | 4.69e-30 | 7.08e-29 | covariate |
| atypical symptomatic | 3.11e+00 | 1.99e+00 | 4.88e+00 | 1.14e+00 | 2.29e-01 | 4.96e+00 | 7.23e-07 | 6.55e-06 | covariate |
| sexW | 1.10e+00 | 8.73e-01 | 1.38e+00 | 9.18e-02 | 1.16e-01 | 7.89e-01 | 4.30e-01 | 1.00e+00 | confounding |

DAG: Associated features

```
odds_ratios_covariates <- sum_table_test_results %>% filter(type == "covariate") %>% as.data.frame()

par(mgp = c(2.5,1,0), font.lab = 2, mfrow=c(1,1), mar = c(10.1, 4.1, 0.2, 0.2))
plot(odds_ratios_covariates$odds_ratio,
     ylim = c(0, 6), pch = 20, cex = 3, xaxt = "n", xlab = "",  ylab = "odds ratio")
sapply(seq_len(nrow(odds_ratios_covariates)), function(i){
  arrows(x0=i, y0=odds_ratios_covariates$`2.5 %`[i], x1=i, y1=odds_ratios_covariates$`97.5 %`[i], code=3, col="black", lwd=2, angle=90, length=0.1)
})
axis(side = 1, at = seq_len(nrow(odds_ratios_covariates)), labels = odds_ratios_covariates %>% pull(Feature), las = 2, mgp = c(3, 0.75, 0))
abline(h = 1, lty = 2)
abline(v = cumsum(unlist(lapply(list_sum_tables, function(tab) nrow(tab %>% filter(type %in% "covariate"))))) + 0.5, lty = 2)
```

```
svg(file.path(plt_dir, "Odds_ratio_models.svg"), width = 15, height = 5)
odds_ratios_covariates <- sum_table_test_results %>% filter(type == "covariate") %>% as.data.frame()

par(mgp = c(2.5,1,0), font.lab = 2, mfrow=c(1,1), mar = c(10.1, 4.1, 0.2, 0.2))
plot(odds_ratios_covariates$odds_ratio,
     ylim = c(0, 6), pch = 20, cex = 3, xaxt = "n", xlab = "",  ylab = "odds ratio")
sapply(seq_len(nrow(odds_ratios_covariates)), function(i){
  arrows(x0=i, y0=odds_ratios_covariates$`2.5 %`[i], x1=i, y1=odds_ratios_covariates$`97.5 %`[i], code=3, col="black", lwd=2, angle=90, length=0.1)
})
axis(side = 1, at = seq_len(nrow(odds_ratios_covariates)), labels = odds_ratios_covariates %>% pull(Feature), las = 2, mgp = c(3, 0.75, 0))
abline(h = 1, lty = 2)
abline(v = cumsum(unlist(lapply(list_sum_tables, function(tab) nrow(tab %>% filter(type %in% "covariate"))))) + 0.5, lty = 2)
dev.off()

pdf(file.path(plt_dir, "Odds_ratio_models.pdf"), width = 15, height = 5)
odds_ratios_covariates <- sum_table_test_results %>% filter(type == "covariate") %>% as.data.frame()

par(mgp = c(2.5,1,0), font.lab = 2, mfrow=c(1,1), mar = c(10.1, 4.1, 0.2, 0.2))
plot(odds_ratios_covariates$odds_ratio,
     ylim = c(0, 6), pch = 20, cex = 3, xaxt = "n", xlab = "",  ylab = "odds ratio")
sapply(seq_len(nrow(odds_ratios_covariates)), function(i){
  arrows(x0=i, y0=odds_ratios_covariates$`2.5 %`[i], x1=i, y1=odds_ratios_covariates$`97.5 %`[i], code=3, col="black", lwd=2, angle=90, length=0.1)
})
axis(side = 1, at = seq_len(nrow(odds_ratios_covariates)), labels = odds_ratios_covariates %>% pull(Feature), las = 2, mgp = c(3, 0.75, 0))
abline(h = 1, lty = 2)
abline(v = cumsum(unlist(lapply(list_sum_tables, function(tab) nrow(tab %>% filter(type %in% "covariate"))))) + 0.5, lty = 2)
dev.off()
```

# Associated factors with symptom response

- reduce analysis only to two categories
  - “0” -> atypical or no symptoms
  - “1” -> typical symptoms

```
plt_dir <- file.path("plots/lasso_symptoms")
res_dir <- file.path("results/lasso_symptoms")

if(!dir.exists(plt_dir)){
  dir.create(plt_dir)
}

if(!dir.exists(res_dir)){
  dir.create(res_dir)
}

df <- df %>% dplyr::select( -`test result`, -manufacturer, -`viral load`,) %>% 
              mutate(symptoms = if_else(symptoms == 1, true = 1, false = 0))
```

## Logistic regression

**ASSUMPTION OF THE ABSENCE OF MULTICOLLINEARITY**

Logistic regression requires there to be little or no
multicollinearity among the independent variables. This means that the
independent variables should not be too highly correlated with each
other.

```
df_box_tid <- df %>% dplyr::mutate(omicron = as.numeric(omicron),
                           `vaccination status` = as.numeric(`vaccination status`),
                           sexW = if_else(sex == "W", true = 1, false = 0)) %>%
                               # age = if_else(age == 0, true = 0.5, false = age)) %>%
                dplyr::select(-sex)

png(filename = file.path(plt_dir, paste0("Scatter_all_pairs.png")),units="px", width=5000, height=5000, res=300)
pairs(df_box_tid %>% dplyr::select(-ID, -symptoms), lower.panel = panel.cor, pch = 20)
dev.off()

pairs(df_box_tid %>% dplyr::select(-ID, -symptoms), lower.panel = panel.cor, pch = 20)
```

**ASSUMPTION OF LINEARITY OF INDEPENDENT VARIABLES AND LOG
ODDS**

Check with Box-Tidwell test with transformed continuous variables

```
lreg <- glm(symptoms ~ age + ageTrans + sexW  + `vaccination status` + omicron, 
            data = df_box_tid %>% mutate(ageTrans = age * log(age)), 
            family=binomial(link="logit"))

as.data.frame(summary(lreg)$coefficients) %>% 
  as_tibble(rownames = "Feature") %>% 
  mutate(across(where(is.double), 
                ~ format(.x, digits = 2, scientific = T))) %>% 
  knitr::kable()
```

| Feature | Estimate | Std. Error | z value | Pr(>|z|) |
| --- | --- | --- | --- | --- |
| (Intercept) | 2.6e+00 | 2.8e-01 | 9.2e+00 | 5.0e-20 |
| age | -1.6e-01 | 3.2e-02 | -5.1e+00 | 4.1e-07 |
| ageTrans | 3.4e-02 | 6.8e-03 | 5.0e+00 | 6.4e-07 |
| sexW | 8.0e-02 | 1.2e-01 | 6.9e-01 | 4.9e-01 |
| `vaccination status` | -7.4e-01 | 1.6e-01 | -4.8e+00 | 2.0e-06 |
| omicron | -7.5e-01 | 1.8e-01 | -4.3e+00 | 2.0e-05 |

**AGE and AGETRANS show significant p values**

**Visual check of age shows linear relationship**

```
logit_model <- glm(symptoms ~ age + sexW + `vaccination status` + omicron, 
                   data = df_box_tid, 
                   family=binomial(link="logit"))

logodds <- logit_model$linear.predictors

plot.dat <- data.frame(logodds = logodds, age = df_box_tid$age)
ggplot(plot.dat, aes(x=age, y=logodds)) + geom_point()
```

**–> Remove age from logistic regression analysis
<–**

## Pairwise comparison: Age and Vaccination status

```
df4test <- df_box_tid %>% 
        dplyr::rename(vaccination_status = `vaccination status`) %>% 
        dplyr::mutate(vaccination_status = factor(if_else(vaccination_status == 1, true = "vaccinated", false = "not vaccinated"),
                                                  levels = c("vaccinated", "not vaccinated")))

wt_res <- wilcox.test(x = df4test %>% dplyr::filter(vaccination_status %in% "vaccinated") %>% pull(age),
                      y = df4test %>% dplyr::filter(vaccination_status %in% "not vaccinated") %>% pull(age))

df4test %>% 
  ggplot2::ggplot(ggplot2::aes(x = vaccination_status, y = age, fill = vaccination_status)) +
  ggplot2::stat_boxplot(geom = "errorbar", lwd = 1, width = 0.5, show.legend = FALSE) +
  ggplot2::geom_boxplot(color = "black", lwd = 1, show.legend = FALSE, outlier.size = 0) +
  ggbeeswarm::geom_quasirandom(alpha = 0.8, width = 0.4, pch = 20, size = 1) +
  ggplot2::theme_bw() +
  ggsci::scale_fill_nejm() +
  ggplot2::theme(axis.text = ggplot2::element_text(size = 14, colour = "black"),
                 axis.title = ggplot2::element_text(face = "bold", size = 12),
                 legend.title = ggplot2::element_blank(),
                 legend.text = ggplot2::element_text(size = 12),
                 legend.position = "none") +
  ggplot2::xlab("vaccination status") +
  ggplot2::ylab("Age") +
  ggsignif::geom_signif(comparisons = list(c("vaccinated", "not vaccinated")),
                        annotation = format(wt_res$p.value, scientific = T, digits = 2),
                        tip_length = c(0.2, 0.04)) + 
  ggplot2::scale_y_continuous(expand = expansion(mult = c(0.05, .1)))
```

## Pairwise comparison: age and symptoms

```
df4test <- df_box_tid %>% 
        dplyr::mutate(symptoms = factor(if_else(symptoms == 0, true = "no", false = "yes"), levels = c("yes", "no")))

 wt_res <- wilcox.test(x = df4test %>% dplyr::filter(symptoms %in% "yes") %>% pull(age),
                       y = df4test %>% dplyr::filter(symptoms %in% "no") %>% pull(age))

df4test %>% 
  ggplot2::ggplot(ggplot2::aes(x = symptoms, y = age, fill = symptoms)) +
  ggplot2::stat_boxplot(geom = "errorbar", lwd = 1, width = 0.5, show.legend = FALSE) +
  ggplot2::geom_boxplot(color = "black", lwd = 1, show.legend = FALSE, outlier.size = 0) +
  ggbeeswarm::geom_quasirandom(alpha = 0.8, width = 0.4, pch = 20, size = 1) +
  ggplot2::theme_bw() +
  ggsci::scale_fill_bmj() +
  ggplot2::theme(axis.text = ggplot2::element_text(size = 14, colour = "black"),
                 axis.title = ggplot2::element_text(face = "bold", size = 12),
                 legend.title = ggplot2::element_blank(),
                 legend.text = ggplot2::element_text(size = 12),
                 legend.position = "none") +
  ggplot2::xlab("Typical symptoms") +
  ggplot2::ylab("Age") +
  ggsignif::geom_signif(comparisons = list(c("yes", "no")),
                        annotation = format(wt_res$p.value, scientific = T, digits = 2),
                        tip_length = c(0.2, 0.04)) + 
  ggplot2::scale_y_continuous(expand = expansion(mult = c(0.05, .1)))
```

## Data set with all variants

```
df <- df %>% dplyr::select(-age)

y <- df %>% pull(symptoms)
X <- df %>% dplyr::select(-ID, -symptoms) %>%
            dplyr::mutate(sex = factor(sex, levels = c("M", "W")),
                          `vaccination status` = as.factor(as.numeric(`vaccination status`)))
X_lasso <- X %>% mutate(across(where(is.factor), .fns = as.integer)) %>% as.matrix()

## 3. 10-fold cross validation to estimate lambda

set.seed(10)
lambdas2try <- exp(seq(-6, 2, length.out = 120))
lasso_cv <- glmnet::cv.glmnet(x = X_lasso, y = y, family = "binomial", alpha = 1, nfolds = 10, lambda = lambdas2try,
                              intercept = FALSE, standardize = TRUE)


model_all_lambdas <- glmnet::glmnet(x = X_lasso, y = y, family = "binomial", alpha = 1, nfolds = 10, lambda = lambdas2try)
par(mfcol = c(1,2), mar=c(5,4.,2.,1)+0.1, font.lab = 2)
plot(lasso_cv)
put.fig.letter(label="a", location="topleft", font=1, cex = 1.5)
matplot(log(model_all_lambdas$lambda), t(as.matrix(model_all_lambdas$beta)), type = "l", lty = rep(c(1,2), each = 9), col = RColorBrewer::brewer.pal(6,"Dark2"), lwd = 2,
        xlab = parse(text = ("Log(lambda)")), ylab = "Value of coefficients")
legend("topright", lty = rep(c(1,2), each = 9), col = rep(RColorBrewer::brewer.pal(6,"Dark2"), 2) , lwd = 1.5, cex = .7,
       legend = rownames(model_all_lambdas$beta), bty = "n")
put.fig.letter(label="b", location="topleft", font=1, cex = 1.5)
```

```
pdf(file.path(plt_dir, "LASSO_REGRESSION.pdf"), height = 5)
par(mfcol = c(1,2), mar=c(5,4.,2.,1)+0.1, font.lab = 2)
plot(lasso_cv)
put.fig.letter(label="a", location="topleft", font=1, cex = 1.5)
matplot(log(model_all_lambdas$lambda), t(as.matrix(model_all_lambdas$beta)), type = "l", lty = rep(c(1,2), each = 9), col = RColorBrewer::brewer.pal(6,"Dark2"), lwd = 2,
        xlab = parse(text = ("Log(lambda)")), ylab = "Value of coefficients")
legend("topright", lty = rep(c(1,2), each = 9), col = rep(RColorBrewer::brewer.pal(6,"Dark2"), 2) , lwd = 1.5, cex = .7,
       legend = rownames(model_all_lambdas$beta), bty = "n")
put.fig.letter(label="b", location="topleft", font=1, cex = 1.5)
dev.off()
```

quartz\_off\_screen 2

## Perform lasso for minimal lambda

```
model_all_lambdas <- glmnet::glmnet(x = X_lasso, y = y, family = "binomial", alpha = 1, nfolds = 10, lambda = lasso_cv$lambda.min)

lambda_cv <- lasso_cv$lambda.min
lasso_best <- broom::tidy(lasso_cv)[lasso_cv$index,] %>% dplyr::rename(MSE = estimate)
lasso_best %>% knitr::kable(digits = 3)
```

| lambda | MSE | std.error | conf.low | conf.high | nzero |
| --- | --- | --- | --- | --- | --- |
| 0.003 | 1.281 | 0.011 | 1.270 | 1.291 | 3 |
| 0.036 | 1.290 | 0.011 | 1.279 | 1.301 | 2 |

## ROC lasso regression

```
## Extract variables unequal zero after lasso
lasso_vars_gt_zero <- rownames(model_all_lambdas$beta)[as.matrix(model_all_lambdas$beta)[,1] != 0]
```

## Final logistic regression based on selected features

### Odds ratios

sex, vaccination status, omicron

```
df_logit <- df %>% dplyr::select(symptoms, matches(paste0(lasso_vars_gt_zero, collapse = "|"))) %>%
            dplyr::mutate(sex = factor(sex, levels = c("M", "W")),
                          `vaccination status` = as.factor(as.numeric(`vaccination status`)),
                          symptoms = factor(symptoms))

# Perform final logistic regression with shrinked data set
glm_full <- glm(symptoms ~ .,data = df_logit, family=binomial(link='logit'))

df_coefs <- as.data.frame(summary(glm_full)$coefficients) %>% as_tibble(rownames = "Feature")

odds_ratio <- data.frame(odds_ratio = exp(coefficients(glm_full)[-1])) %>% as_tibble(rownames = "Feature")
odds_ratio_ci <- data.frame(exp(confint.default(glm_full)[-1,]), check.names = F) %>% as_tibble(rownames = "Feature")

# Store odds ratios and p values of lasso regression

odds_ratio %>% inner_join(odds_ratio_ci) %>% inner_join(df_coefs) %>%
  writexl::write_xlsx(path = file.path(res_dir, "lasso_coefs_odds_ratios.xlsx"))

odds_ratio_ci <- odds_ratio_ci %>% as.data.frame()

# Plot odds ratios and CI

pdf(file.path(plt_dir, "odds_ratio.pdf"), width = 20, height = 7)
par(mgp = c(2.5,1,0), font.lab = 2, mfrow=c(1,1), mar = c(4.1, 4.1, 0.2, 0.2))
plot(seq_len(nrow(odds_ratio)), odds_ratio %>% pull(odds_ratio),
     ylim = c(0, 2.5), pch = 20, cex = 3, xaxt = "n", xlab = "coefficients",  ylab = "odds ratio")
sapply(seq_len(nrow(odds_ratio)), function(i){
  arrows(x0=i, y0=odds_ratio_ci[i,2], x1=i, y1=odds_ratio_ci[i,3], code=3, col="black", lwd=2, angle=90, length=0.25)
})
axis(side = 1, at = seq_len(nrow(odds_ratio)), labels = odds_ratio %>% pull(Feature))
abline(h = 1, lty = 2)
dev.off()

# Write data into excel

sample_sizes_df <- df_logit %>% 
                    dplyr::select(matches(paste0(lasso_vars_gt_zero[!lasso_vars_gt_zero %in% c("age", "viral load")], collapse = "|"))) %>% 
                    mutate(omicron = factor(omicron)) %>%
  tidyr::pivot_longer(cols = matches("*"), names_to = "feature", values_to = "value", values_transform = list(value = as.character)) %>%
  group_by(feature, value) %>% count() %>%
  group_by(feature) %>%
  mutate(rel = n/sum(n))
  
writexl::write_xlsx(sample_sizes_df, path = file.path(res_dir, "sample_sizes_for_features.xlsx"))

par(mgp = c(2.5,1,0), font.lab = 2, mfrow=c(1,1), mar = c(4.1, 4.1, 0.2, 0.2))
plot(seq_len(nrow(odds_ratio)), odds_ratio %>% pull(odds_ratio),
     ylim = c(0, 2.5), pch = 20, cex = 3, xaxt = "n", xlab = "coefficients",  ylab = "odds ratio")
sapply(seq_len(nrow(odds_ratio)), function(i){
  arrows(x0=i, y0=odds_ratio_ci[i,2], x1=i, y1=odds_ratio_ci[i,3], code=3, col="black", lwd=2, angle=90, length=0.25)
})
axis(side = 1, at = seq_len(nrow(odds_ratio)), labels = odds_ratio %>% pull(Feature))
abline(h = 1, lty = 2)
```

```
padj_df <- data.frame(Feature = df_coefs$Feature, 
                      p.adj = p.adjust(df_coefs$`Pr(>|z|)`, method = "BY"))

sum_odds_ratio_table <- odds_ratio %>% 
                        inner_join(odds_ratio_ci) %>% 
                        inner_join(df_coefs) %>%
                        inner_join(padj_df)

sum_odds_ratio_table %>% 
  mutate(across(where(is.double), 
                ~ format(.x, digits = 3, scientific = TRUE))) %>% 
  knitr::kable()
```

| Feature | odds\_ratio | 2.5 % | 97.5 % | Estimate | Std. Error | z value | Pr(>|z|) | p.adj |
| --- | --- | --- | --- | --- | --- | --- | --- | --- |
| sexW | 1.09e+00 | 8.73e-01 | 1.36e+00 | 8.51e-02 | 1.13e-01 | 7.54e-01 | 4.51e-01 | 9.39e-01 |
| `vaccination status`2 | 2.93e-01 | 2.31e-01 | 3.71e-01 | -1.23e+00 | 1.21e-01 | -1.02e+01 | 2.77e-24 | 2.31e-23 |
| omicron | 5.93e-01 | 4.28e-01 | 8.21e-01 | -5.23e-01 | 1.66e-01 | -3.14e+00 | 1.68e-03 | 4.67e-03 |

### Group sizes

```
sample_sizes_df %>% mutate(across(where(is.double), ~ round(.x, digits = 2))) %>% 
knitr::kable()
```

| feature | value | n | rel |
| --- | --- | --- | --- |
| omicron | 0 | 205 | 0.14 |
| omicron | 1 | 1267 | 0.86 |
| sex | M | 797 | 0.54 |
| sex | W | 675 | 0.46 |
| vaccination status | 1 | 494 | 0.34 |
| vaccination status | 2 | 978 | 0.66 |

## Logistic regression: vaccination status (Model 1)

```
glm_full_vaccs <- glm(symptoms ~ `vaccination status`, data = df_logit, family=binomial(link='logit'))

df_coefs_vaccs <- as.data.frame(summary(glm_full_vaccs)$coefficients) %>% as_tibble(rownames = "Feature")

odds_ratio_vaccs <- data.frame(odds_ratio = exp(coefficients(glm_full_vaccs)[-1])) %>% as_tibble(rownames = "Feature")
odds_ratio_ci_vaccs <- data.frame(exp(confint.default(glm_full_vaccs)), check.names = F)[-1,] %>% as_tibble(rownames = "Feature")

# Store odds ratios and p values of lasso regression

odds_ratio_vaccs %>% inner_join(odds_ratio_ci_vaccs) %>% inner_join(df_coefs_vaccs) %>%
  writexl::write_xlsx(path = file.path(res_dir, "lasso_coefs_odds_ratios_vaccs.xlsx"))

odds_ratio_ci_vaccs <- odds_ratio_ci_vaccs %>% as.data.frame()

# Plot odds ratios and CI

pdf(file.path(plt_dir, "odds_ratio.pdf"), width = 20, height = 7)
par(mgp = c(2.5,1,0), font.lab = 2, mfrow=c(1,1), mar = c(4.1, 4.1, 0.2, 0.2))
plot(seq_len(nrow(odds_ratio_vaccs)), odds_ratio_vaccs %>% pull(odds_ratio),
     ylim = c(-1,2.5), pch = 20, cex = 3, xaxt = "n", xlab = "coefficients",  ylab = "odds ratio")
if(nrow(odds_ratio_vaccs) == 1){
     
  arrows(x0=1, y0=odds_ratio_ci_vaccs[1,2], x1=1, y1=odds_ratio_ci_vaccs[1,3], code=3, col="black", lwd=2, angle=90, length=0.25)
     
}else{

  sapply(seq_len(nrow(odds_ratio_vaccs)), function(i){
    arrows(x0=i, y0=odds_ratio_ci_vaccs[i,2], x1=i, y1=odds_ratio_ci_vaccs[i,2], code=3, col="black", lwd=2, angle=90, length=0.25)
  })
}
axis(side = 1, at = seq_len(nrow(odds_ratio_vaccs)), labels = odds_ratio_vaccs %>% pull(Feature))
abline(h = 1, lty = 2)
dev.off()
```

```
padj_df_vaccs <- data.frame(Feature = df_coefs_vaccs$Feature, 
                      p.adj = p.adjust(df_coefs_vaccs$`Pr(>|z|)`, method = "BY"))

sum_odds_ratio_table_vaccs <- odds_ratio_vaccs %>% 
                        inner_join(odds_ratio_ci_vaccs) %>% 
                        inner_join(df_coefs_vaccs) %>%
                        inner_join(padj_df_vaccs)

sum_odds_ratio_table_vaccs %>% 
  mutate(across(where(is.double), 
                ~ format(.x, digits = 3, scientific = TRUE))) %>% 
  knitr::kable()
```

| Feature | odds\_ratio | 2.5 % | 97.5 % | Estimate | Std. Error | z value | Pr(>|z|) | p.adj |
| --- | --- | --- | --- | --- | --- | --- | --- | --- |
| `vaccination status`2 | 2.62e-01 | 2.09e-01 | 3.29e-01 | -1.34e+00 | 1.16e-01 | -1.15e+01 | 8.3e-31 | 2.49e-30 |

## Logistic regression: Omicron (Model 2)

```
glm_full_omicron <- glm(symptoms ~ omicron, data = df_logit, family=binomial(link='logit'))

df_coefs_omicron <- as.data.frame(summary(glm_full_omicron)$coefficients) %>% as_tibble(rownames = "Feature")

odds_ratio_omicron <- data.frame(odds_ratio = exp(coefficients(glm_full_omicron)[-1])) %>% as_tibble(rownames = "Feature")
odds_ratio_ci_omicron <- data.frame(exp(confint.default(glm_full_omicron)), check.names = F)[-1,] %>% as_tibble(rownames = "Feature")

# Store odds ratios and p values of lasso regression

odds_ratio_omicron %>% inner_join(odds_ratio_ci_omicron) %>% inner_join(df_coefs_omicron) %>%
  writexl::write_xlsx(path = file.path(res_dir, "lasso_coefs_odds_ratios_omicron.xlsx"))

odds_ratio_ci_omicron <- odds_ratio_ci_omicron %>% as.data.frame()

# Plot odds ratios and CI

pdf(file.path(plt_dir, "odds_ratio.pdf"), width = 20, height = 7)
par(mgp = c(2.5,1,0), font.lab = 2, mfrow=c(1,1), mar = c(4.1, 4.1, 0.2, 0.2))
plot(seq_len(nrow(odds_ratio_omicron)), odds_ratio_omicron %>% pull(odds_ratio),
     ylim = c(-1,2.5), pch = 20, cex = 3, xaxt = "n", xlab = "coefficients",  ylab = "odds ratio")
if(nrow(odds_ratio_omicron) == 1){
     
  arrows(x0=1, y0=odds_ratio_ci_omicron[1,2], x1=1, y1=odds_ratio_ci_omicron[1,3], code=3, col="black", lwd=2, angle=90, length=0.25)
     
}else{

  sapply(seq_len(nrow(odds_ratio_omicron)), function(i){
    arrows(x0=i, y0=odds_ratio_ci_omicron[i,2], x1=i, y1=odds_ratio_ci_omicron[i,2], code=3, col="black", lwd=2, angle=90, length=0.25)
  })
}
axis(side = 1, at = seq_len(nrow(odds_ratio_omicron)), labels = odds_ratio_omicron %>% pull(Feature))
abline(h = 1, lty = 2)
dev.off()
```

```
padj_df_omicron <- data.frame(Feature = df_coefs_omicron$Feature, 
                      p.adj = p.adjust(df_coefs_omicron$`Pr(>|z|)`, method = "BY"))

sum_odds_ratio_table_omicron <- odds_ratio_omicron %>% 
                        inner_join(odds_ratio_ci_omicron) %>% 
                        inner_join(df_coefs_omicron) %>%
                        inner_join(padj_df_omicron)

sum_odds_ratio_table_omicron %>% 
  mutate(across(where(is.double), 
                ~ format(.x, digits = 3, scientific = TRUE))) %>% 
  knitr::kable()
```

| Feature | odds\_ratio | 2.5 % | 97.5 % | Estimate | Std. Error | z value | Pr(>|z|) | p.adj |
| --- | --- | --- | --- | --- | --- | --- | --- | --- |
| omicron | 3.7e-01 | 2.73e-01 | 5e-01 | -9.95e-01 | 1.54e-01 | -6.44e+00 | 1.16e-10 | 3.49e-10 |

## Logistic regression: Sex (Model 3)

```
glm_full_sex <- glm(symptoms ~ sex, data = df_logit, family=binomial(link='logit'))

df_coefs_sex <- as.data.frame(summary(glm_full_sex)$coefficients) %>% as_tibble(rownames = "Feature")

odds_ratio_sex <- data.frame(odds_ratio = exp(coefficients(glm_full_sex)[-1])) %>% as_tibble(rownames = "Feature")
odds_ratio_ci_sex <- data.frame(exp(confint.default(glm_full_sex)), check.names = F)[-1,] %>% as_tibble(rownames = "Feature")

# Store odds ratios and p values of lasso regression

odds_ratio_sex %>% inner_join(odds_ratio_ci_sex) %>% inner_join(df_coefs_sex) %>%
  writexl::write_xlsx(path = file.path(res_dir, "lasso_coefs_odds_ratios_sex.xlsx"))

odds_ratio_ci_sex <- odds_ratio_ci_sex %>% as.data.frame()

# Plot odds ratios and CI

pdf(file.path(plt_dir, "odds_ratio.pdf"), width = 20, height = 7)
par(mgp = c(2.5,1,0), font.lab = 2, mfrow=c(1,1), mar = c(4.1, 4.1, 0.2, 0.2))
plot(seq_len(nrow(odds_ratio_sex)), odds_ratio_sex %>% pull(odds_ratio),
     ylim = c(-1,2.5), pch = 20, cex = 3, xaxt = "n", xlab = "coefficients",  ylab = "odds ratio")
if(nrow(odds_ratio_sex) == 1){
     
  arrows(x0=1, y0=odds_ratio_ci_sex[1,2], x1=1, y1=odds_ratio_ci_sex[1,3], code=3, col="black", lwd=2, angle=90, length=0.25)
     
}else{

  sapply(seq_len(nrow(odds_ratio_sex)), function(i){
    arrows(x0=i, y0=odds_ratio_ci_sex[i,2], x1=i, y1=odds_ratio_ci_sex[i,2], code=3, col="black", lwd=2, angle=90, length=0.25)
  })
}
axis(side = 1, at = seq_len(nrow(odds_ratio_sex)), labels = odds_ratio_sex %>% pull(Feature))
abline(h = 1, lty = 2)
dev.off()
```

```
padj_df_sex <- data.frame(Feature = df_coefs_sex$Feature, 
                      p.adj = p.adjust(df_coefs_sex$`Pr(>|z|)`, method = "BY"))

sum_odds_ratio_table_sex <- odds_ratio_sex %>% 
                        inner_join(odds_ratio_ci_sex) %>% 
                        inner_join(df_coefs_sex) %>%
                        inner_join(padj_df_sex)

sum_odds_ratio_table_sex %>% 
  mutate(across(where(is.double), 
                ~ format(.x, digits = 3, scientific = TRUE))) %>% 
  knitr::kable()
```

| Feature | odds\_ratio | 2.5 % | 97.5 % | Estimate | Std. Error | z value | Pr(>|z|) | p.adj |
| --- | --- | --- | --- | --- | --- | --- | --- | --- |
| sexW | 1.1e+00 | 8.9e-01 | 1.35e+00 | 9.29e-02 | 1.07e-01 | 8.68e-01 | 3.85e-01 | 5.78e-01 |

# Summarized models

```
sum_odds_ratio_table_vaccs <- sum_odds_ratio_table_vaccs %>% 
                                arrange(Feature)
                                
sum_odds_ratio_table_omicron <- sum_odds_ratio_table_omicron %>% 
                                arrange(Feature) 
                                
sum_odds_ratio_table_sex <- sum_odds_ratio_table_sex %>% 
                                arrange(Feature) 
                                
sum_odds_ratio_table <- sum_odds_ratio_table %>% 
                                arrange(Feature) 
                                                           
sum_table_test_results <- do.call("rbind", list(sum_odds_ratio_table,
                                                sum_odds_ratio_table_vaccs,
                                                sum_odds_ratio_table_omicron,
                                                sum_odds_ratio_table_sex))

sum_table_test_results <- sum_table_test_results %>% dplyr::rename("p value" = `Pr(>|z|)`)
sum_table_test_results$p.adj <- p.adjust(sum_table_test_results$`p value`, method = "BY")

index_line <- cumsum(c(nrow(sum_odds_ratio_table),
                       nrow(sum_odds_ratio_table_vaccs), 
                       nrow(sum_odds_ratio_table_omicron), 
                       nrow(sum_odds_ratio_table_sex))) + 1
                       
knitr::kable(sum_table_test_results %>% 
              mutate(across(where(is.double), 
                    ~ format(.x, digits = 3, scientific = TRUE)))) %>%
  kable_paper("striped", full_width = F) %>%
   kableExtra::pack_rows(group_label = "Model 1 (full)", start_row = 1, end_row = index_line[1]-1, hline_after = T, indent = F) %>%
   kableExtra::pack_rows(group_label = "Model 2 (vaccination status)", start_row = index_line[1], end_row = index_line[2], hline_after = T, indent = F) %>% 
   kableExtra::pack_rows(group_label = "Model 3 (omicron)", start_row = index_line[2], end_row = index_line[3]-1, hline_after = T, indent = F) %>%
   kableExtra::pack_rows(group_label = "Model 3 (sex)", start_row = index_line[3], end_row = index_line[4]-1, hline_after = T, indent = F) %>%
   kable_styling() %>%
   row_spec(c(1:3, index_line[1], index_line[2], index_line[3]), 
              bold=T, hline_after = T)
```

| Feature | odds\_ratio | 2.5 % | 97.5 % | Estimate | Std. Error | z value | p value | p.adj |
| --- | --- | --- | --- | --- | --- | --- | --- | --- |
| **Model 1 (full)** | | | | | | | | |
| `vaccination status`2 | 2.93e-01 | 2.31e-01 | 3.71e-01 | -1.23e+00 | 1.21e-01 | -1.02e+01 | 2.77e-24 | 2.04e-23 |
| omicron | 5.93e-01 | 4.28e-01 | 8.21e-01 | -5.23e-01 | 1.66e-01 | -3.14e+00 | 1.68e-03 | 6.18e-03 |
| sexW | 1.09e+00 | 8.73e-01 | 1.36e+00 | 8.51e-02 | 1.13e-01 | 7.54e-01 | 4.51e-01 | 1.00e+00 |
| **Model 2 (vaccination status)** | | | | | | | | |
| `vaccination status`2 | 2.62e-01 | 2.09e-01 | 3.29e-01 | -1.34e+00 | 1.16e-01 | -1.15e+01 | 8.30e-31 | 1.22e-29 |
| **Model 3 (omicron)** | | | | | | | | |
| omicron | 3.70e-01 | 2.73e-01 | 5.00e-01 | -9.95e-01 | 1.54e-01 | -6.44e+00 | 1.16e-10 | 5.70e-10 |
| **Model 3 (sex)** | | | | | | | | |
| sexW | 1.10e+00 | 8.90e-01 | 1.35e+00 | 9.29e-02 | 1.07e-01 | 8.68e-01 | 3.85e-01 | 1.00e+00 |

```
odds_ratios_covariates <- sum_table_test_results %>% as.data.frame()

par(mgp = c(2.5,1,0), font.lab = 2, mfrow=c(1,1), mar = c(10.1, 4.1, 0.2, 0.2))
plot(odds_ratios_covariates$odds_ratio,
     ylim = c(0, 2.5), pch = 20, cex = 3, xaxt = "n", xlab = "",  ylab = "odds ratio")
sapply(seq_len(nrow(odds_ratios_covariates)), function(i){
  arrows(x0=i, y0=odds_ratios_covariates$`2.5 %`[i], x1=i, y1=odds_ratios_covariates$`97.5 %`[i], code=3, col="black", lwd=2, angle=90, length=0.25)
})
axis(side = 1, at = seq_len(nrow(odds_ratios_covariates)), labels = odds_ratios_covariates %>% pull(Feature), las = 2, mgp = c(3, 0.75, 0))
abline(h = 1, lty = 2)
abline(v = c(1:3, 5, 7) + 0.5, lty = 2)
```

# Factors influencing test result of RDT (only first participation)

```
plt_dir <- file.path("plots/lasso_test_results_single_participation")
res_dir <- file.path("results/lasso_test_results_single_participation")

if(!dir.exists(plt_dir)){
  dir.create(plt_dir)
}

if(!dir.exists(res_dir)){
  dir.create(res_dir)
}

df <- readr::read_csv2("data/single_participation_data.csv") %>% 
      mutate(manufacturer = as.factor(manufacturer)) %>% 
      dplyr::rename(sex = gender)
```

**ASSUMPTION OF THE ABSENCE OF MULTICOLLINEARITY**

```
df_box_tid <- df %>% dplyr::mutate(symptoms1 = if_else(symptoms == 1, true = 1, false = 0),
                           symptoms2 = if_else(symptoms == 2, true = 1, false = 0),
                           omicron = as.numeric(omicron),
                           `vaccination status` = as.numeric(`vaccination status`),
                           `viral load` = as.numeric( `viral load`),
                           sexW = if_else(sex == "W", true = 1, false = 0)) %>%
                               # age = if_else(age == 0, true = 0.5, false = age)) %>%
                dplyr::select(-symptoms, -sex)

png(filename = file.path(plt_dir, paste0("Scatter_all_pairs.png")),units="px", width=5000, height=5000, res=300)
pairs(df_box_tid %>% dplyr::select(-ID, -`test result`), lower.panel = panel.cor, pch = 20)
dev.off()

pairs(df_box_tid %>% dplyr::select(-ID, -`test result`), lower.panel = panel.cor, pch = 20)
```

**ASSUMPTION OF LINEARITY OF INDEPENDENT VARIABLES AND LOG
ODDS**

Check with Box-Tidwell test with transformed continuous variables

```
lreg <- glm(`test result` ~ age + ageTrans + `viral load` + viral_loadTrans + sexW + symptoms1 + symptoms2 + `vaccination status` + omicron + manufacturer, 
            data = df_box_tid %>% mutate(ageTrans = age * log(age),
                                         viral_loadTrans = `viral load` * log(`viral load`)), 
            family=binomial(link="logit"))

as.data.frame(summary(lreg)$coefficients) %>% 
  as_tibble(rownames = "Feature") %>% 
  mutate(across(where(is.double), 
                ~ format(.x, digits = 2, scientific = T))) %>% 
knitr::kable()
```

| Feature | Estimate | Std. Error | z value | Pr(>|z|) |
| --- | --- | --- | --- | --- |
| (Intercept) | -6.0e+00 | 2.3e+00 | -2.6e+00 | 1.0e-02 |
| age | 2.2e-04 | 4.7e-02 | 4.8e-03 | 1.0e+00 |
| ageTrans | 1.3e-03 | 9.9e-03 | 1.3e-01 | 9.0e-01 |
| `viral load` | 6.5e-01 | 1.1e+00 | 6.1e-01 | 5.4e-01 |
| viral\_loadTrans | 1.1e-01 | 3.8e-01 | 2.9e-01 | 7.7e-01 |
| sexW | 1.1e-01 | 1.8e-01 | 6.4e-01 | 5.2e-01 |
| symptoms1 | 8.9e-01 | 2.0e-01 | 4.4e+00 | 1.1e-05 |
| symptoms2 | 2.3e-01 | 3.4e-01 | 6.6e-01 | 5.1e-01 |
| `vaccination status` | -4.0e-01 | 2.3e-01 | -1.7e+00 | 9.0e-02 |
| omicron | -1.5e-01 | 2.6e-01 | -5.8e-01 | 5.6e-01 |
| manufacturer2 | 3.9e-01 | 3.9e-01 | 1.0e+00 | 3.2e-01 |
| manufacturer3 | 1.5e-01 | 3.5e-01 | 4.2e-01 | 6.7e-01 |

## Adult data set with all variants

```
y <- df %>% pull(`test result`)
X <- df %>% dplyr::select(-ID, -`test result`) %>%
            dplyr::mutate(symptoms = factor(symptoms),# + 1, levels = 1:3),
                          sex = factor(sex, levels = c("M", "W")),
                          `vaccination status` = as.factor(as.numeric(`vaccination status`)),
                          manufacturer = factor(manufacturer))
X_lasso <- X %>% mutate(across(where(is.factor), .fns = as.integer)) %>% as.matrix()

## 3. 10-fold cross validation to estimate lambda

set.seed(10)
lambdas2try <- exp(seq(-6, 2, length.out = 120))
lasso_cv <- glmnet::cv.glmnet(x = X_lasso, y = y, family = "binomial", alpha = 1, nfolds = 10, lambda = lambdas2try,
                              intercept = FALSE, standardize = TRUE)


model_all_lambdas <- glmnet::glmnet(x = X_lasso, y = y, family = "binomial", alpha = 1, nfolds = 10, lambda = lambdas2try)
par(mfcol = c(1,2), mar=c(5,4.,2.,1)+0.1, font.lab = 2)
plot(lasso_cv)
put.fig.letter(label="a", location="topleft", font=1, cex = 1.5)
matplot(log(model_all_lambdas$lambda), t(as.matrix(model_all_lambdas$beta)), type = "l", lty = rep(c(1,2), each = 9), col = RColorBrewer::brewer.pal(6,"Dark2"), lwd = 2,
        xlab = parse(text = ("Log(lambda)")), ylab = "Value of coefficients")
legend("topright", lty = rep(c(1,2), each = 9), col = rep(RColorBrewer::brewer.pal(6,"Dark2"), 2) , lwd = 1.5, cex = .7,
       legend = rownames(model_all_lambdas$beta), bty = "n")
put.fig.letter(label="b", location="topleft", font=1, cex = 1.5)
```

```
pdf(file.path(plt_dir, "LASSO_REGRESSION.pdf"), height = 5)
par(mfcol = c(1,2), mar=c(5,4.,2.,1)+0.1, font.lab = 2)
plot(lasso_cv)
put.fig.letter(label="a", location="topleft", font=1, cex = 1.5)
matplot(log(model_all_lambdas$lambda), t(as.matrix(model_all_lambdas$beta)), type = "l", lty = rep(c(1,2), each = 9), col = RColorBrewer::brewer.pal(6,"Dark2"), lwd = 2,
        xlab = parse(text = ("Log(lambda)")), ylab = "Value of coefficients")
legend("topright", lty = rep(c(1,2), each = 9), col = rep(RColorBrewer::brewer.pal(6,"Dark2"), 2) , lwd = 1.5, cex = .7,
       legend = rownames(model_all_lambdas$beta), bty = "n")
put.fig.letter(label="b", location="topleft", font=1, cex = 1.5)
dev.off()
```

## Perform lasso for minimal lambda

```
model_all_lambdas <- glmnet::glmnet(x = X_lasso, y = y, family = "binomial", alpha = 1, nfolds = 10, lambda = lasso_cv$lambda.min)

lambda_cv <- lasso_cv$lambda.min
lasso_best <- broom::tidy(lasso_cv)[lasso_cv$index,] %>% dplyr::rename(MSE = estimate)
lasso_best %>% knitr::kable(digits = 3)
```

| lambda | MSE | std.error | conf.low | conf.high | nzero |
| --- | --- | --- | --- | --- | --- |
| 0.003 | 0.976 | 0.027 | 0.949 | 1.003 | 7 |
| 0.032 | 1.001 | 0.022 | 0.979 | 1.023 | 5 |

## ROC lasso regression

## Final logistic regression based on selected features

```
## Extract varaibles unequal zero after lasso
lasso_vars_gt_zero <- rownames(model_all_lambdas$beta)[as.matrix(model_all_lambdas$beta)[,1] != 0]
```

### Odds ratios

viral load, age, sex, symptoms, vaccination status, omicron

```
df_logit <- df %>% dplyr::select(`test result`, matches(paste0(lasso_vars_gt_zero, collapse = "|"))) %>%
            dplyr::mutate(symptoms = factor(symptoms),
                          sex = factor(sex, levels = c("M", "W")),
                          `vaccination status` = as.factor(as.numeric(`vaccination status`)))

# Perform final logistic regression with shrinked data set
glm_full <- glm(`test result` ~ .,data = df_logit, family=binomial(link='logit'))

df_coefs <- as.data.frame(summary(glm_full)$coefficients) %>% as_tibble(rownames = "Feature")

odds_ratio <- data.frame(odds_ratio = exp(coefficients(glm_full)[-1])) %>% as_tibble(rownames = "Feature")
odds_ratio_ci <- data.frame(exp(confint.default(glm_full)[-1,]), check.names = F) %>% as_tibble(rownames = "Feature")

# Store odds ratios and p values of lasso regression

odds_ratio %>% inner_join(odds_ratio_ci) %>% inner_join(df_coefs) %>%
  writexl::write_xlsx(path = file.path(res_dir, "lasso_coefs_odds_ratios.xlsx"))

odds_ratio_ci <- odds_ratio_ci %>% as.data.frame()

# Plot odds ratios and CI

pdf(file.path(plt_dir, "odds_ratio.pdf"), width = 20, height = 7)
par(mgp = c(2.5,1,0), font.lab = 2, mfrow=c(1,1), mar = c(4.1, 4.1, 0.2, 0.2))
plot(seq_len(nrow(odds_ratio)), odds_ratio %>% pull(odds_ratio),
     ylim = c(-1,5), pch = 20, cex = 3, xaxt = "n", xlab = "coefficients",  ylab = "odds ratio")
sapply(seq_len(nrow(odds_ratio)), function(i){
  arrows(x0=i, y0=odds_ratio_ci[i,2], x1=i, y1=odds_ratio_ci[i,3], code=3, col="black", lwd=2, angle=90, length=0.25)
})
axis(side = 1, at = seq_len(nrow(odds_ratio)), labels = odds_ratio %>% pull(Feature))
abline(h = 1, lty = 2)
dev.off()

# Write data into excel

sample_sizes_df <- df_logit %>% 
                    dplyr::select(matches(paste0(lasso_vars_gt_zero[!lasso_vars_gt_zero %in% c("age", "viral load")], collapse = "|"))) %>% 
                    mutate(omicron = factor(omicron)) %>%
  tidyr::pivot_longer(cols = matches("*"), names_to = "feature", values_to = "value", values_transform = list(value = as.character)) %>%
  group_by(feature, value) %>% count() %>%
  group_by(feature) %>%
  mutate(rel = n/sum(n))
  
writexl::write_xlsx(sample_sizes_df, path = file.path(res_dir, "sample_sizes_for_features.xlsx"))

par(mgp = c(2.5,1,0), font.lab = 2, mfrow=c(1,1), mar = c(4.1, 4.1, 0.2, 0.2))
plot(seq_len(nrow(odds_ratio)), odds_ratio %>% pull(odds_ratio),
     ylim = c(-1,5), pch = 20, cex = 3, xaxt = "n", xlab = "coefficients",  ylab = "odds ratio")
sapply(seq_len(nrow(odds_ratio)), function(i){
  arrows(x0=i, y0=odds_ratio_ci[i,2], x1=i, y1=odds_ratio_ci[i,3], code=3, col="black", lwd=2, angle=90, length=0.25)
})
axis(side = 1, at = seq_len(nrow(odds_ratio)), labels = odds_ratio %>% pull(Feature))
abline(h = 1, lty = 2)
```

**Odds ratio table**

```
padj_df <- data.frame(Feature = df_coefs$Feature, 
                      p.adj = p.adjust(df_coefs$`Pr(>|z|)`, method = "BY"))

sum_odds_ratio_table <- odds_ratio %>% 
                        inner_join(odds_ratio_ci) %>% 
                        inner_join(df_coefs) %>%
                        inner_join(padj_df)

sum_odds_ratio_table %>% 
  mutate(across(where(is.double), 
                ~ format(.x, digits = 3, scientific = TRUE))) %>% 
  knitr::kable()
```

| Feature | odds\_ratio | 2.5 % | 97.5 % | Estimate | Std. Error | z value | Pr(>|z|) | p.adj |
| --- | --- | --- | --- | --- | --- | --- | --- | --- |
| `viral load` | 2.64e+00 | 2.33e+00 | 3.00e+00 | 9.72e-01 | 6.49e-02 | 1.50e+01 | 9.94e-51 | 2.16e-49 |
| age | 1.00e+00 | 9.98e-01 | 1.01e+00 | 4.56e-03 | 3.46e-03 | 1.32e+00 | 1.88e-01 | 8.17e-01 |
| sexW | 1.06e+00 | 7.55e-01 | 1.49e+00 | 6.03e-02 | 1.74e-01 | 3.46e-01 | 7.29e-01 | 1.00e+00 |
| symptoms1 | 2.42e+00 | 1.66e+00 | 3.54e+00 | 8.85e-01 | 1.94e-01 | 4.56e+00 | 5.05e-06 | 3.66e-05 |
| symptoms2 | 1.32e+00 | 6.86e-01 | 2.56e+00 | 2.81e-01 | 3.36e-01 | 8.37e-01 | 4.03e-01 | 1.00e+00 |
| `vaccination status`2 | 6.54e-01 | 4.25e-01 | 1.01e+00 | -4.24e-01 | 2.20e-01 | -1.93e+00 | 5.41e-02 | 2.94e-01 |
| omicron | 8.89e-01 | 5.51e-01 | 1.43e+00 | -1.18e-01 | 2.44e-01 | -4.82e-01 | 6.30e-01 | 1.00e+00 |

### Group sizes

```
sample_sizes_df %>% 
  mutate(across(where(is.double), ~ round(.x, digits = 2))) %>% 
  knitr::kable()
```

| feature | value | n | rel |
| --- | --- | --- | --- |
| omicron | 0 | 176 | 0.18 |
| omicron | 1 | 828 | 0.82 |
| sex | M | 522 | 0.52 |
| sex | W | 482 | 0.48 |
| symptoms | 0 | 505 | 0.50 |
| symptoms | 1 | 425 | 0.42 |
| symptoms | 2 | 74 | 0.07 |
| vaccination status | 1 | 408 | 0.41 |
| vaccination status | 2 | 596 | 0.59 |

## Logistic regression on viral load (Model 1)

```
# Perform final logistic regression with shrinked data set
glm_load <- glm(`test result` ~  `viral load` + age + sex + `vaccination status`, data = df_logit, family = binomial(link='logit'))

df_coefs_load <- as.data.frame(summary(glm_load)$coefficients) %>% as_tibble(rownames = "Feature")

odds_ratio_load <- data.frame(odds_ratio = exp(coefficients(glm_load)[-1])) %>% as_tibble(rownames = "Feature")
odds_ratio_ci_load <- data.frame(exp(confint.default(glm_load)[-1,]), check.names = F) %>% as_tibble(rownames = "Feature")

# Store odds ratios and p values of lasso regression

odds_ratio_load %>% inner_join(odds_ratio_ci_load) %>% inner_join(df_coefs) %>%
  writexl::write_xlsx(path = file.path(res_dir, "lasso_coefs_odds_ratios_load.xlsx"))

odds_ratio_ci_load <- odds_ratio_ci_load %>% as.data.frame()

# Plot odds ratios and CI

pdf(file.path(plt_dir, "odds_ratio_load.pdf"), width = 20, height = 7)
par(mgp = c(2.5,1,0), font.lab = 2, mfrow=c(1,1), mar = c(4.1, 4.1, 0.2, 0.2))
plot(seq_len(nrow(odds_ratio_load)), odds_ratio_load %>% pull(odds_ratio),
     ylim = c(-1,6), pch = 20, cex = 3, xaxt = "n", xlab = "coefficients",  ylab = "odds ratio")
sapply(seq_len(nrow(odds_ratio_load)), function(i){
  arrows(x0=i, y0=odds_ratio_ci_load[i,2], x1=i, y1=odds_ratio_ci_load[i,3], code=3, col="black", lwd=2, angle=90, length=0.25)
})
axis(side = 1, at = seq_len(nrow(odds_ratio_load)), labels = odds_ratio_load %>% pull(Feature))
abline(h = 1, lty = 2)
dev.off()

par(mgp = c(2.5,1,0), font.lab = 2, mfrow=c(1,1), mar = c(4.1, 4.1, 0.2, 0.2))
plot(seq_len(nrow(odds_ratio_load)), odds_ratio_load %>% pull(odds_ratio),
     ylim = c(-1,6), pch = 20, cex = 3, xaxt = "n", xlab = "coefficients",  ylab = "odds ratio")
sapply(seq_len(nrow(odds_ratio_load)), function(i){
  arrows(x0=i, y0=odds_ratio_ci_load[i,2], x1=i, y1=odds_ratio_ci_load[i,3], code=3, col="black", lwd=2, angle=90, length=0.25)
})
axis(side = 1, at = seq_len(nrow(odds_ratio_load)), labels = odds_ratio_load %>% pull(Feature))
abline(h = 1, lty = 2)
```

**Odds ratio table**

```
padj_df <- data.frame(Feature = df_coefs_load$Feature, 
                      p.adj = p.adjust(df_coefs_load$`Pr(>|z|)`, method = "BY"))

sum_odds_ratio_table_load <- odds_ratio_load %>% 
                        inner_join(odds_ratio_ci_load) %>% 
                        inner_join(df_coefs_load) %>%
                        inner_join(padj_df)

sum_odds_ratio_table_load %>% 
  mutate(across(where(is.double), 
                ~ format(.x, digits = 3, scientific = TRUE))) %>% 
  knitr::kable()
```

| Feature | odds\_ratio | 2.5 % | 97.5 % | Estimate | Std. Error | z value | Pr(>|z|) | p.adj |
| --- | --- | --- | --- | --- | --- | --- | --- | --- |
| `viral load` | 2.73e+00 | 2.41e+00 | 3.10e+00 | 1.01e+00 | 6.36e-02 | 1.58e+01 | 2.47e-56 | 2.82e-55 |
| age | 1.00e+00 | 9.98e-01 | 1.01e+00 | 4.39e-03 | 3.28e-03 | 1.34e+00 | 1.81e-01 | 5.18e-01 |
| sexW | 1.08e+00 | 7.68e-01 | 1.50e+00 | 7.26e-02 | 1.72e-01 | 4.23e-01 | 6.72e-01 | 1.00e+00 |
| `vaccination status`2 | 4.81e-01 | 3.27e-01 | 7.07e-01 | -7.32e-01 | 1.97e-01 | -3.72e+00 | 2.01e-04 | 7.67e-04 |

## Logistic regression on symptoms (Model 3)

```
# Perform final logistic regression with shrinked data set
glm_symp <- glm(`test result` ~  symptoms + age + sex + `vaccination status`, data = df_logit, family = binomial(link='logit'))

df_coefs_symp <- as.data.frame(summary(glm_symp)$coefficients) %>% as_tibble(rownames = "Feature")

odds_ratio_symp <- data.frame(odds_ratio = exp(coefficients(glm_symp)[-1])) %>% as_tibble(rownames = "Feature")
odds_ratio_ci_symp <- data.frame(exp(confint.default(glm_symp)[-1,]), check.names = F) %>% as_tibble(rownames = "Feature")

# Store odds ratios and p values of lasso regression

odds_ratio_symp %>% inner_join(odds_ratio_ci_symp) %>% inner_join(df_coefs) %>%
  writexl::write_xlsx(path = file.path(res_dir, "lasso_coefs_odds_ratios_symp.xlsx"))

odds_ratio_ci_symp <- odds_ratio_ci_symp %>% as.data.frame()

# Plot odds ratios and CI

pdf(file.path(plt_dir, "odds_ratio_symp.pdf"), width = 20, height = 7)
par(mgp = c(2.5,1,0), font.lab = 2, mfrow=c(1,1), mar = c(4.1, 4.1, 0.2, 0.2))
plot(seq_len(nrow(odds_ratio_symp)), odds_ratio_symp %>% pull(odds_ratio),
     ylim = c(-1,6), pch = 20, cex = 3, xaxt = "n", xlab = "coefficients",  ylab = "odds ratio")
sapply(seq_len(nrow(odds_ratio_symp)), function(i){
  arrows(x0=i, y0=odds_ratio_ci_symp[i,2], x1=i, y1=odds_ratio_ci_symp[i,3], code=3, col="black", lwd=2, angle=90, length=0.25)
})
axis(side = 1, at = seq_len(nrow(odds_ratio_symp)), labels = odds_ratio_symp %>% pull(Feature))
abline(h = 1, lty = 2)
dev.off()

par(mgp = c(2.5,1,0), font.lab = 2, mfrow=c(1,1), mar = c(4.1, 4.1, 0.2, 0.2))
plot(seq_len(nrow(odds_ratio_symp)), odds_ratio_symp %>% pull(odds_ratio),
     ylim = c(-1,6), pch = 20, cex = 3, xaxt = "n", xlab = "coefficients",  ylab = "odds ratio")
sapply(seq_len(nrow(odds_ratio_symp)), function(i){
  arrows(x0=i, y0=odds_ratio_ci_symp[i,2], x1=i, y1=odds_ratio_ci_symp[i,3], code=3, col="black", lwd=2, angle=90, length=0.25)
})
axis(side = 1, at = seq_len(nrow(odds_ratio_symp)), labels = odds_ratio_symp %>% pull(Feature))
abline(h = 1, lty = 2)
```

**Odds ratio table**

```
padj_df <- data.frame(Feature = df_coefs_symp$Feature, 
                      p.adj = p.adjust(df_coefs_symp$`Pr(>|z|)`, method = "BY"))

sum_odds_ratio_table_symp <- odds_ratio_symp %>% 
                        inner_join(odds_ratio_ci_symp) %>% 
                        inner_join(df_coefs_symp) %>%
                        inner_join(padj_df)

sum_odds_ratio_table_symp %>% 
  mutate(across(where(is.double), 
                ~ format(.x, digits = 3, scientific = TRUE))) %>% 
  knitr::kable()
```

| Feature | odds\_ratio | 2.5 % | 97.5 % | Estimate | Std. Error | z value | Pr(>|z|) | p.adj |
| --- | --- | --- | --- | --- | --- | --- | --- | --- |
| symptoms1 | 3.89e+00 | 2.89e+00 | 5.23e+00 | 1.36e+00 | 1.51e-01 | 8.97e+00 | 3.07e-19 | 4.51e-18 |
| symptoms2 | 2.99e+00 | 1.80e+00 | 4.95e+00 | 1.09e+00 | 2.58e-01 | 4.25e+00 | 2.15e-05 | 1.05e-04 |
| age | 1.01e+00 | 1.00e+00 | 1.01e+00 | 6.24e-03 | 2.64e-03 | 2.36e+00 | 1.81e-02 | 6.67e-02 |
| sexW | 1.03e+00 | 7.84e-01 | 1.35e+00 | 2.66e-02 | 1.38e-01 | 1.93e-01 | 8.47e-01 | 1.00e+00 |
| `vaccination status`2 | 8.87e-01 | 6.45e-01 | 1.22e+00 | -1.20e-01 | 1.63e-01 | -7.39e-01 | 4.60e-01 | 1.00e+00 |

## Logistic regression on omicron infection (Model 3)

```
# Perform final logistic regression with shrinked data set
glm_omic <- glm(`test result` ~  omicron + age + sex, data = df_logit, family = binomial(link='logit'))

df_coefs_omic <- as.data.frame(summary(glm_omic)$coefficients) %>% as_tibble(rownames = "Feature")

odds_ratio_omic <- data.frame(odds_ratio = exp(coefficients(glm_omic)[-1])) %>% as_tibble(rownames = "Feature")
odds_ratio_ci_omic <- data.frame(exp(confint.default(glm_omic)[-1,]), check.names = F) %>% as_tibble(rownames = "Feature")

# Store odds ratios and p values of lasso regression

odds_ratio_omic %>% inner_join(odds_ratio_ci_omic) %>% inner_join(df_coefs) %>%
  writexl::write_xlsx(path = file.path(res_dir, "lasso_coefs_odds_ratios_omic.xlsx"))

odds_ratio_ci_omic <- odds_ratio_ci_omic %>% as.data.frame()

# Plot odds ratios and CI

pdf(file.path(plt_dir, "odds_ratio_omic.pdf"), width = 20, height = 7)
par(mgp = c(2.5,1,0), font.lab = 2, mfrow=c(1,1), mar = c(4.1, 4.1, 0.2, 0.2))
plot(seq_len(nrow(odds_ratio_omic)), odds_ratio_omic %>% pull(odds_ratio),
     ylim = c(-1,6), pch = 20, cex = 3, xaxt = "n", xlab = "coefficients",  ylab = "odds ratio")
sapply(seq_len(nrow(odds_ratio_omic)), function(i){
  arrows(x0=i, y0=odds_ratio_ci_omic[i,2], x1=i, y1=odds_ratio_ci_omic[i,3], code=3, col="black", lwd=2, angle=90, length=0.25)
})
axis(side = 1, at = seq_len(nrow(odds_ratio_omic)), labels = odds_ratio_omic %>% pull(Feature))
abline(h = 1, lty = 2)
dev.off()

par(mgp = c(2.5,1,0), font.lab = 2, mfrow=c(1,1), mar = c(4.1, 4.1, 0.2, 0.2))
plot(seq_len(nrow(odds_ratio_omic)), odds_ratio_omic %>% pull(odds_ratio),
     ylim = c(-1,6), pch = 20, cex = 3, xaxt = "n", xlab = "coefficients",  ylab = "odds ratio")
sapply(seq_len(nrow(odds_ratio_omic)), function(i){
  arrows(x0=i, y0=odds_ratio_ci_omic[i,2], x1=i, y1=odds_ratio_ci_omic[i,3], code=3, col="black", lwd=2, angle=90, length=0.25)
})
axis(side = 1, at = seq_len(nrow(odds_ratio_omic)), labels = odds_ratio_omic %>% pull(Feature))
abline(h = 1, lty = 2)
```

**Odds ratio table**

```
padj_df <- data.frame(Feature = df_coefs_omic$Feature, 
                      p.adj = p.adjust(df_coefs_omic$`Pr(>|z|)`, method = "BY"))

sum_odds_ratio_table_omic <- odds_ratio_omic %>% 
                        inner_join(odds_ratio_ci_omic) %>% 
                        inner_join(df_coefs_omic) %>%
                        inner_join(padj_df)

sum_odds_ratio_table_omic %>% 
  mutate(across(where(is.double), 
                ~ format(.x, digits = 3, scientific = TRUE))) %>% 
  knitr::kable()
```

| Feature | odds\_ratio | 2.5 % | 97.5 % | Estimate | Std. Error | z value | Pr(>|z|) | p.adj |
| --- | --- | --- | --- | --- | --- | --- | --- | --- |
| omicron | 9.03e-01 | 6.47e-01 | 1.26e+00 | -1.02e-01 | 1.70e-01 | -5.99e-01 | 5.49e-01 | 1e+00 |
| age | 1.00e+00 | 9.97e-01 | 1.01e+00 | 1.40e-03 | 2.25e-03 | 6.21e-01 | 5.35e-01 | 1e+00 |
| sexW | 1.00e+00 | 7.75e-01 | 1.30e+00 | 1.96e-03 | 1.31e-01 | 1.49e-02 | 9.88e-01 | 1e+00 |

# Summarized models

```
sum_odds_ratio_table_load <- sum_odds_ratio_table_load %>% 
                                mutate(type = if_else(Feature %in% c("`viral load`", "symptoms1", "symptoms2"), 
                                                      true = "covariate", false = "confounding"),
                                       type = factor(type, levels = c("covariate", "confounding"))) %>% 
                                arrange(type, Feature)

sum_odds_ratio_table_omic <- sum_odds_ratio_table_omic %>% 
                                mutate(type = if_else(Feature %in% c("`viral load`", "symptoms1", "symptoms2", "omicron"), 
                                                      true = "covariate", false = "confounding"),
                                       type = factor(type, levels = c("covariate", "confounding"))) %>% 
                                arrange(type, Feature) 

sum_odds_ratio_table_symp <- sum_odds_ratio_table_symp %>% 
                                mutate(type = if_else(Feature %in% c("`viral load`", "symptoms1", "symptoms2"), 
                                                      true = "covariate", false = "confounding"),
                                       type = factor(type, levels = c("covariate", "confounding"))) %>% 
                                arrange(type, Feature) %>%
                                mutate(Feature = case_when(Feature == "symptoms1" ~ "typical symptomatic", 
                                                           Feature == "symptoms2" ~ "atypical symptomatic",
                                                           TRUE ~ Feature))
  
sum_table_test_results <- do.call("rbind", list(sum_odds_ratio_table_load, 
                                                sum_odds_ratio_table_symp,
                                                sum_odds_ratio_table_omic))

sum_table_test_results <- sum_table_test_results %>% dplyr::rename("p value" = `Pr(>|z|)`)
sum_table_test_results$p.adj <- p.adjust(sum_table_test_results$`p value`, method = "BY")

index_line <- cumsum(c(nrow(sum_odds_ratio_table_load), nrow(sum_odds_ratio_table_symp), nrow(sum_odds_ratio_table_omic))) + 1
knitr::kable(sum_table_test_results %>% 
              mutate(across(where(is.double), 
                    ~ format(.x, digits = 3, scientific = TRUE)))) %>%
  kable_paper("striped", full_width = F) %>%
   kableExtra::pack_rows(group_label = "Model 1 (viral load)", 1, index_line[1],hline_after = T, indent = F) %>% 
   kableExtra::pack_rows(group_label = "Model 2 (symptoms)", start_row = index_line[1], end_row = index_line[2], hline_after = T, indent = F) %>% 
   kableExtra::pack_rows(group_label = "Model 3 (omicron)", start_row = index_line[2], end_row = index_line[3]-1, hline_after = T, indent = F) %>%
   kable_styling()%>%
   row_spec(c(1, index_line[1], index_line[1] +1, index_line[2]),bold=T,hline_after = T)
```

| Feature | odds\_ratio | 2.5 % | 97.5 % | Estimate | Std. Error | z value | p value | p.adj | type |
| --- | --- | --- | --- | --- | --- | --- | --- | --- | --- |
| **Model 1 (viral load)** | | | | | | | | | |
| `viral load` | 2.73e+00 | 2.41e+00 | 3.10e+00 | 1.01e+00 | 6.36e-02 | 1.58e+01 | 2.47e-56 | 9.21e-55 | covariate |
| `vaccination status`2 | 4.81e-01 | 3.27e-01 | 7.07e-01 | -7.32e-01 | 1.97e-01 | -3.72e+00 | 2.01e-04 | 1.88e-03 | confounding |
| age | 1.00e+00 | 9.98e-01 | 1.01e+00 | 4.39e-03 | 3.28e-03 | 1.34e+00 | 1.81e-01 | 1.00e+00 | confounding |
| sexW | 1.08e+00 | 7.68e-01 | 1.50e+00 | 7.26e-02 | 1.72e-01 | 4.23e-01 | 6.72e-01 | 1.00e+00 | confounding |
| **Model 2 (symptoms)** | | | | | | | | | |
| typical symptomatic | 3.89e+00 | 2.89e+00 | 5.23e+00 | 1.36e+00 | 1.51e-01 | 8.97e+00 | 3.07e-19 | 5.71e-18 | covariate |
| atypical symptomatic | 2.99e+00 | 1.80e+00 | 4.95e+00 | 1.09e+00 | 2.58e-01 | 4.25e+00 | 2.15e-05 | 2.66e-04 | covariate |
| `vaccination status`2 | 8.87e-01 | 6.45e-01 | 1.22e+00 | -1.20e-01 | 1.63e-01 | -7.39e-01 | 4.60e-01 | 1.00e+00 | confounding |
| age | 1.01e+00 | 1.00e+00 | 1.01e+00 | 6.24e-03 | 2.64e-03 | 2.36e+00 | 1.81e-02 | 1.35e-01 | confounding |
| sexW | 1.03e+00 | 7.84e-01 | 1.35e+00 | 2.66e-02 | 1.38e-01 | 1.93e-01 | 8.47e-01 | 1.00e+00 | confounding |
| **Model 3 (omicron)** | | | | | | | | | |
| omicron | 9.03e-01 | 6.47e-01 | 1.26e+00 | -1.02e-01 | 1.70e-01 | -5.99e-01 | 5.49e-01 | 1.00e+00 | covariate |
| age | 1.00e+00 | 9.97e-01 | 1.01e+00 | 1.40e-03 | 2.25e-03 | 6.21e-01 | 5.35e-01 | 1.00e+00 | confounding |
| sexW | 1.00e+00 | 7.75e-01 | 1.30e+00 | 1.96e-03 | 1.31e-01 | 1.49e-02 | 9.88e-01 | 1.00e+00 | confounding |

```
odds_ratios_covariates <- sum_table_test_results %>% filter(type == "covariate") %>% as.data.frame()

par(mgp = c(2.5,1,0), font.lab = 2, mfrow=c(1,1), mar = c(4.1, 4.1, 0.2, 0.2))
plot(odds_ratios_covariates$odds_ratio,
     ylim = c(0, 6), pch = 20, cex = 3, xaxt = "n", xlab = "coefficients",  ylab = "odds ratio")
sapply(seq_len(nrow(odds_ratios_covariates)), function(i){
  arrows(x0=i, y0=odds_ratios_covariates$`2.5 %`[i], x1=i, y1=odds_ratios_covariates$`97.5 %`[i], code=3, col="black", lwd=2, angle=90, length=0.25)
})
axis(side = 1, at = seq_len(nrow(odds_ratios_covariates)), labels = odds_ratios_covariates %>% pull(Feature))
abline(h = 1, lty = 2)
```
